# The haplotype-resolved telomere-to-telomere carnation (*Dianthus caryophyllus*) genome reveals the correlation between genome architecture and gene expression

**DOI:** 10.1093/hr/uhad244

**Published:** 2023-11-27

**Authors:** Lan Lan, Luhong Leng, Weichao Liu, Yonglin Ren, Wayne Reeve, Xiaopeng Fu, Zhiqiang Wu, Xiaoni Zhang

**Affiliations:** Shenzhen Branch, Guangdong Laboratory of Lingnan Modern Agriculture, Key Laboratory of Synthetic Biology, Laboratory of the Ministry of Agriculture and Rural Affairs, Agricultural Genomics Institute at Shenzhen, Chinese Academy of Agricultural Sciences, Shenzhen 518124, China; College of Science, Health, Engineering and Education, Murdoch University, Murdoch 6150, Western Australia, Australia; Kunpeng Institute of Modern Agriculture at Foshan, Shenzhen Branch, Guangdong Laboratory of Lingnan Modern Agriculture, Agricultural Genomics Institute at Shenzhen, Chinese Academy of Agricultural Sciences, Shenzhen 518124, China; Shenzhen Branch, Guangdong Laboratory of Lingnan Modern Agriculture, Key Laboratory of Synthetic Biology, Laboratory of the Ministry of Agriculture and Rural Affairs, Agricultural Genomics Institute at Shenzhen, Chinese Academy of Agricultural Sciences, Shenzhen 518124, China; Kunpeng Institute of Modern Agriculture at Foshan, Shenzhen Branch, Guangdong Laboratory of Lingnan Modern Agriculture, Agricultural Genomics Institute at Shenzhen, Chinese Academy of Agricultural Sciences, Shenzhen 518124, China; Shenzhen Branch, Guangdong Laboratory of Lingnan Modern Agriculture, Key Laboratory of Synthetic Biology, Laboratory of the Ministry of Agriculture and Rural Affairs, Agricultural Genomics Institute at Shenzhen, Chinese Academy of Agricultural Sciences, Shenzhen 518124, China; Kunpeng Institute of Modern Agriculture at Foshan, Shenzhen Branch, Guangdong Laboratory of Lingnan Modern Agriculture, Agricultural Genomics Institute at Shenzhen, Chinese Academy of Agricultural Sciences, Shenzhen 518124, China; Key Laboratory of Horticultural Plant Biology, College of Horticulture and Forestry Sciences, Huazhong Agricultural University, Wuhan, 430070, China; College of Science, Health, Engineering and Education, Murdoch University, Murdoch 6150, Western Australia, Australia; College of Science, Health, Engineering and Education, Murdoch University, Murdoch 6150, Western Australia, Australia; Key Laboratory of Horticultural Plant Biology, College of Horticulture and Forestry Sciences, Huazhong Agricultural University, Wuhan, 430070, China; Shenzhen Branch, Guangdong Laboratory of Lingnan Modern Agriculture, Key Laboratory of Synthetic Biology, Laboratory of the Ministry of Agriculture and Rural Affairs, Agricultural Genomics Institute at Shenzhen, Chinese Academy of Agricultural Sciences, Shenzhen 518124, China; Kunpeng Institute of Modern Agriculture at Foshan, Shenzhen Branch, Guangdong Laboratory of Lingnan Modern Agriculture, Agricultural Genomics Institute at Shenzhen, Chinese Academy of Agricultural Sciences, Shenzhen 518124, China; Kunpeng Institute of Modern Agriculture at Foshan, Shenzhen Branch, Guangdong Laboratory of Lingnan Modern Agriculture, Agricultural Genomics Institute at Shenzhen, Chinese Academy of Agricultural Sciences, Shenzhen 518124, China

## Abstract

Carnation (*Dianthus caryophyllus*) is one of the most valuable commercial flowers, due to its richness of color and form, and its excellent storage and vase life. The diverse demands of the market require faster breeding in carnations. A full understanding of carnations is therefore required to guide the direction of breeding. Hence, we assembled the haplotype-resolved gap-free carnation genome of the variety ‘Baltico’, which is the most common white standard variety worldwide. Based on high-depth HiFi, ultra-long nanopore, and Hi-C sequencing data, we assembled the telomere-to-telomere (T2T) genome to be 564 479 117 and 568 266 215 bp for the two haplotypes Hap1 and Hap2, respectively. This T2T genome exhibited great improvement in genome assembly and annotation results compared with the former version. The improvements were seen when different approaches to evaluation were used. Our T2T genome first informs the analysis of the telomere and centromere region, enabling us to speculate about specific centromere characteristics that cannot be identified by high-order repeats in carnations. We analyzed allele-specific expression in three tissues and the relationship between genome architecture and gene expression in the haplotypes. This demonstrated that the length of the genes, coding sequences, and introns, the exon numbers and the transposable element insertions correlate with gene expression ratios and levels. The insertions of transposable elements repress expression in gene regulatory networks in carnation. This gap-free finished T2T carnation genome provides a valuable resource to illustrate the genome characteristics and for functional genomics analysis in further studies and molecular breeding.

## Introduction

Carnations (*Dianthus caryophyllus* L.) belong to the family Caryophyllaceae and are a major ornamental plant species found throughout the world. Due to their colorful flowers and abundant forms, they are widely used as cut flowers and potted and yard flowers, and in the landscaping of flower beds that are loved by people all over the world. Although there are already many varieties of carnation on the market, there is a strong demand from consumers and growers for new cultivars with specific characteristics. Consumers expect cut flowers to have a range of colors, rich fragrance, and long vase life. Growers expect them to be disease resistant and to bloom continuously. So far, growers have been using interspecific and intraspecific hybridization strategies for creating cultivars with diversity and quality of ornamental traits [1, 2]. In recent years, it has been reported that a high-quality genome helps to increase the efficiency of breeding and improvement of quality in plants such as apple [3] and coconut [4].

Assembling a high-quality genome is a common way to gain a better understanding of a species. Researchers today can easily obtain chromosomal-level assemblies using Pacific Biosciences (PacBio) single-molecule real-time (SMRT), Oxford Nanopore Technologies (ONT), and high-throughput chromatin conformation capture (Hi-C) sequencing technology. However, for several years [5] there have been gaps in the genome because of the weakness of the assembly algorithms, sequencing methods, and so on [6]. Most of these gaps exist in tandem repeats and segmental duplications that are difficult to resolve [7]. In the first reported telomere-to-telomere (T2T) human genome, the gaps occurred in regions where there were tandem and complex repeats [8]. These expanded repeat contents and repeat-mediated structural rearrangements provide insight into the evolution of the species and the chromosome structure [9]. The short reads are known to produce low-quality *de novo* genome assemblies, because they cannot span long and complex regions [10]. For this reason, there were many gaps in draft genomes, which reduced the number of genes and overlooked the ‘dark matter’ regions in the genome assembly. The improvements in long-read sequencing technologies, particularly third-generation sequencing technology and assembly algorithms, enabled T2T assembled genomes to be achieved.

A complete T2T genome containing the full genome information of a species, could be seen as a final goal of a genome assembly. It would avoid mapping errors and improve the precision of calling variation; identify genes and genetic information that has been lost; provide more accurate haplotype genome information; and reveal the evolutionary history of centromeres and telomeres [11]. To date, several genomes have been reported as being gap-free or T2T, such as *Arabidopsis* [12], watermelon [13], rice [14], kiwifruit [15], bitter melon [16], *Rhodomyrtus tomentosa* [17], and grapes [18]. However, no T2T genome has been published for carnation. The previously published chromosome-level *D. caryophyllus* ‘Scarlet Queen’ (SQ) genome retains several gaps [19] (Table 1).

**Table 1:** Assembly statistics of the ‘Baltico’ and SQ genomes.

	Initial assembly of ‘Baltico’		Gap-filled T2T assembly of ‘Baltico’		SQ
	Hap1	Hap2	Hap1	Hap2	
Contig number	29	29	15	15	32
Genome size (bp)	563 683 187	564 651 758	564 479 117	568 266 215	636 302 055
N50 (bp)	32 814 548	33 947 117	37 578 261	38 006 131	38 554 967
BUSCO genome (EM)	C, 97.9%; D, 4.6%	C, 97.2%; D, 4.0%	C, 98.0%; D, 4.6%	C, 97.4%; D, 4.6%	C, 95.6%; D, 4.6%
BUSCO genome (EU)	C, 93.8%; D, 5.8%	C, 93.2%; D, 5.5%	C, 93.9%; D, 5.8%	C, 93.7%; D, 6.0%	C, 93.9%; D, 9.2%
LAI	-	-	23.88	24.13	23.36
Gap numbers	14 + 4 (telomere)	14 + 6 (telomere)	0	0	45 + 9 (missed telomeres) + 17 unplaced contigs
Repeat content (bp)	-	-	392 980 145	394 537 150	449 364 514
Repeat content (%)	-	-	69.62	69.43	70.62
Gene numbers	-	-	41 669	40 486	43 925
BUSCO protein (EM)	-	-	C, 97.6%; D, 4.8%	C, 97.6%; D, 4.8%	C, 95.3%; D, 8.0%
BUSCO protein (EU)	-	-	C, 93.7%; D, 6.0%	C, 93.8%; D, 6.2%	C, 91.2%; D, 8.2%

C refers to complete BUSCOs; D refers to complete and duplicated BUSCOs.

Gene expression is one of the key processes of trait formation. Recently many studies found that genetic variation and unbalanced expression of alleles are responsible for trait diversity. An imbalance in mRNA abundance between alleles has been referred to as ‘allele-specific expression’ (ASE). For example, in apple, *MYB110a* encodes a transcription factor regulating anthocyanin biosynthesis, and a transposable element (TE) insertion in the allele *MYB110a* results in ASE [20]. In strawberries, researchers found that specific TE insertion into an allele of *TFL* caused ASE, resulting in a change from flowering once to continuous flowering [21]. Similar findings have been found in other species, such as *Arabidopsis* [22–24] and barley [25].

Genome architecture is the arrangement of functional elements within the genome [26] and can be represented in a linear fashion. It can play a pivotal role in gene regulation [27]. For example, introns have been shown to have multiple effects on expression regulation [28, 29]. The widely distributed TEs among eukaryotic species contribute to genome architecture, and undergo independent expansion [30]. TEs have been reported to be able to regulate the expression of genes in regulatory networks [30], enhancers [31, 32], transcription factor binding sites [33], insulator sequences [34], and repressive elements [35]. Research in apple [3] demonstrated that TE insertion can enhance gene expression and alter the phenotype. In general, intact long terminal repeats (LTRs) harbor promoter and terminator sequences [36], and could be identified more in the completed genome assemblies [37]. TEs have been reported that are ubiquitous in the *D. caryophyllus* genome [19, 38], but remain less discussed. In addition, the lack of complete and accurate genomic data has hampered our studies of genome architecture.

By combining long- and short-read sequencing data with state-of-the-art assembly algorithms, we generated haplotype-resolved T2T genome assemblies of *D. caryophyllus* ‘Baltico’. Based on the gap-free genome, we bridged the gaps in all telomeres and analyzed the ‘dark regions’ within the telomeres and centromeres. We speculate that *D. caryophyllus* may have a unique centromere region. Based on the comprehensive genome architectures of the haplotypes, we investigated the correlation between genome architecture and gene expression. We analyzed the expression patterns of allelic genes and found that 29.28–33.94% of them showed ASE in different tissues between the two haplotypes. We also found specific genome architecture between the haplotypes which could contribute to the ASE. We speculate that the gene, coding sequence (CDS), and intron lengths and exon numbers were correlated with gene expression, and that TEs insertions are widely characterized as repressive elements involved in gene regulatory networks in *D. caryophyllus*.

## Results

### The telomere-to-telomere genome assembly and annotation of *D. caryophyllus* ‘Baltico’

Total sequencing data with 73.45 Gb of high-fidelity (HiFi) data, 57.77 Gb of ultra-long (UL) ONT data, 44.64 Gb of Hi-C data, and 32.3 Gb of short-read data were used for genome assembly. The HiFi data used for genome assembly had an average length of 17 622.2 bp and an average base quality score of 31.1 (Supplementary Data Fig. S1A and B). The UL ONT data used for genome assembly had an average length of 38 018.9 bp and an average base quality score of 11.06 (Supplementary Data Fig. S1C and D). By filtering read lengths shorter than 100 kb, we got a total of 4.04 Gb data with an average length of 124 546.5 bp and average base quality score of 11.04 (Supplementary Data Fig. S1E and F).

Through uniting Graphical Fragment Assembly (GFA) generated by hifiasm, we obtained a high continuous genome graph (Supplementary Data Fig. S2). The primary assembly of ‘Baltico’ revealed the size to be 563 683 187 and 567 007 489 bp for two different haplotypes, hereafter identified as Hap1 and Hap2, respectively, and each assembly contained a total of 29 contigs (Table 1). To evaluate the genome quality precisely, the two different Benchmarking Universal Single-Copy Orthologs (BUSCO) databases, eudicots_odb10 (EU) and embryophyta_odb10 (EM), were used. The primary assembly was of high quality, as revealed by BUSCO evaluation results, for both haplotypes. Complete scores were 97.9% (EM) and 93.8% (EU) for Hap1, 97.2% (EM) and 93.2% (EU) for Hap2 (Supplementary Data Fig. S3A). In addition, low duplication scores of 4.6% (EM) and 5.8% (EU) for Hap1 and 4% (EM) and 5.5% (EU) for Hap2 were revealed. The *k*-mer spectrum plot also reflected the high-quality and haplotype-resolved assembly results (Supplementary Data Fig. S4).

Using the Hi-C data, we successfully oriented the contigs into 15 pseudochromosomes, leaving a total of 28 gaps, of which there were 14 in Hap1 and 14 in Hap2 (Supplementary Data Fig. S5). Furthermore, we detected that four and six telomeres were missing in the Hap1 and Hap2 assemblies, respectively (Table 1).

**Figure 1:**
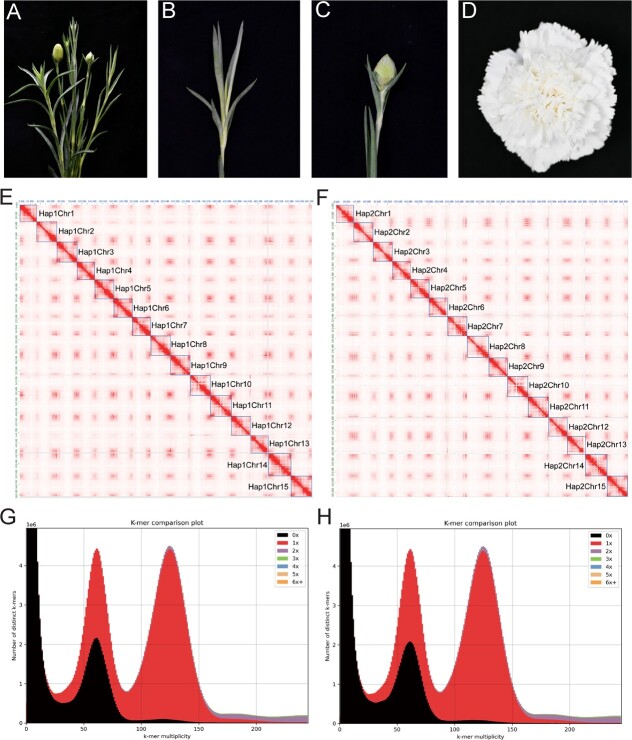
The phenotypes and gap-free genome assembly features of ‘Baltico’. **A**–**D** ‘Baltico’ plant (**A**) and its different tissues: (**B**) shoot, (**C**) flower bud, and (**D**) blooming flower. **E**, **F** Hi-C heat maps of Hap1 (**E**) and Hap2 (**F**). **G**, **H***k*-mer spectrum analysis plots generated by KAT for Hap1 (**F**) and Hap2 (**H**); the black region represents the proportion of *k*-mers present in the HiFi reads but missing in the assemblies.

After closing these gaps by the UL ONT data, we compiled two gap-free ‘Baltico’ haplotypes. For Hap1, the genome size increased from 563 683 187 to 564 479 117 bp and for Hap2 the genome size increased from 564 651 758 to 568 266 215 bp (Table 1, Supplementary Data Table S1). The N50 size increased from 32 814 548 to 37 578 261 bp for Hap1 and from 33 947 117 to 38 006 131 bp for Hap2. Our assembly results were close to the genome survey result, which exhibited a haplotype genome size of 570 416 832 bp (Supplementary Data Fig. S6). Several chromosomes in different haplotypes were highly divergent in length, such as chromosome 8 (Chr8) and Chr14 (Supplementary Data Table S2). The BUSCO evaluation results were improved after the gap-filling process (Table 1). For Hap1, the BUSCO complete value increased from 97.9 to 98.0% (EM) and from 93.8 to 93.9% (EU). For Hap2, the BUSCO complete value increased from 97.2 to 97.4% (EM) and from 93.2 to 93.7% (EU) (Supplementary Data Fig. S3A). The Hi-C heat map showed that errors were absent in the assembly (Fig. 1E and F). The *k*-mer spectral plot revealed that the gap-closing methods had little influence on the haplotype information and the main unique contents were present in the assembly (Fig. 1G and H). The evaluated switch error rate is 1.63% in our assembled genome, and the consensus quality value (QV) evaluated based on the short-sequencing data was 44.916 and 49.470 for Hap1 and Hap2, respectively. In addition, the mapping depth of HiFi data showed that there was less bias (Supplementary Data Fig. S7). The published genetic map 72 L of carnation [39] also shows strong collinearity; the average Pearson correlation coefficient between haplotype genomes was 90.8% (Supplementary Data Figs S8 and S9). Based on these results, we have now compiled two high quality gap-free haplotypes of ‘Baltico’.

A total of 41 669 and 40 486 genes were predicted in the two assembled gap-free genomes of Hap1 and Hap2, respectively, and the BUSCO evaluation results showed high complete scores using both EU and EM (Table 1, Supplementary Data Fig. S3B). The ratio of the number of monoexonic genes to the number of multiexonic genes is 0.28 and 0.27 in Hap1 and Hap2, respectively (Supplementary Data Table S3). Among them, 36 253 (87.00%) and 35 117 (86.74%) genes could be annotated by different databases; moreover, 69.36 and 67.88% of the total genes could be annotated by the Pfam database in Hap1 and Hap2, respectively (Supplementary Data Table S4), indicating reasonable and ideal prediction results [40] and high-quality genome prediction results.

### Comparative analysis and improvements to the ‘Scarlet Queen’ genome

We performed comparative genomics analyses between the published ONT-based SQ genome and the gap-free genomes assembled in this study. There were 45 gaps and 17 unplaced contigs remaining in the SQ genome (Table 1). The larger genome size of SQ may be due to the non-haplotype-aware assembly method, resulting in more redundant sequences. This was reflected by the BUSCO evaluation results as the higher duplication value in both genome assembly and annotation results (Table 1, Supplementary Data Fig. S3A and B). Although the SQ genome predicted more genes than the gap-free genomes, the results of BUSCO evaluation revealed that the SQ genome had a lower quality score (Table 1, Supplementary Data Fig. S3B). Furthermore, the ratio of monoexonic gene numbers to multiexonic gene numbers in SQ is 0.51 which is greater than the normal size of 0.2 (Supplementary Data Table S3). Compared with ‘Baltico’, the SQ genome contained a shorter average gene and CDS length, but longer average exon length; in addition, SQ contained a greater average number of exons per multiexonic gene and a greater number of exons per multiexonic gene (Supplementary Data Table S3). We also found that SQ had a higher proportion of shorter genes and a shorter CDS (Supplementary Data Fig. S10).

**Figure 2:**
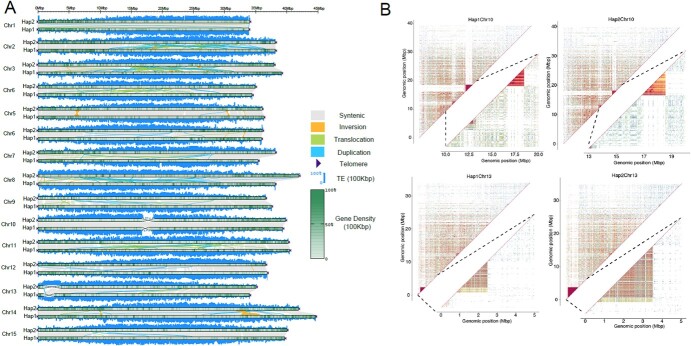
Genome structure of each chromosome and structure of four centromeres in ‘Baltico’ gap-free genomes. **A** Genome features of each chromosome in ‘Baltico’ gap-free haplotypes and collinearity analysis between the haplotypes; bin sizes of different features were all set at 100 kb. **B** Sequence identity heat map of candidate centromere regions for Chr10 and Chr13 in two haplotypes. Cold colors indicate low identity between regions, while warm colors indicate high identity between regions.

We checked the position of the centromeres and telomeres in the two gap-free genomes and the SQ genome. The telomeric repeat region was found at both ends of each chromosome in the two gap-free ‘Baltico’ haplotypes (Fig. 2A, Supplementary Data Table S5), while the SQ genome lacked nine telomeres (Supplementary Data Table S5). The candidate centromere regions were identified by detecting the high-order repeat (HOR) regions, and we detected four candidate centromere regions in the two haplotypes of ‘Baltico’, of which two were found in Chr10 and two were found in Chr13 (Fig. 2B, Supplementary Data Table S6). The candidate centromere size ranges from 995 471 to 2 646 945 bp, and the repeat monomer contains the size of 510 and 32 bp for Chr10 and Chr13 respectively (Fig. 2A and B, Supplementary Data Table S6). We also applied the reads-based approach to identify the candidate centromere region and only detected three candidate regions (Supplementary Data Fig. S11). These results demonstrated the unusual features of the centromere region in *D. caryophyllus*. Furthermore, not only could the centromere region not be identified by HOR in the ‘Baltico’ gap-free genome, but it also could not be identified in the SQ genome.

Comparing the two gap-free haplotypes with the SQ genome, we found strong collinearity between them: the percentage of syntenic region was 80.78 and 78.40% for Hap1 and Hap2 while them compared with SQ, respectively (Supplementary Data Table S7). Several chromosomes, such as Chr10, exhibited extremely strong collinearity between SQ and both gap-free haplotypes. However, in Chr3 the percentage of collinearity regions between SQ and Hap1 was 99.79, while it was only 67.22 between SQ and Hap2. In Chr2, the percentage between SQ and Hap1 was 62.58; however, up to 99.91% of collinearity regions were found between SQ and Hap2. There were 1984 and 1989 structural variations between SQ and Hap1 and Hap2, respectively (Supplementary Data Table S7, Supplementary Data Fig. S12). These findings exhibit the great diversity between the different cultivars.

We also explored the nucleotide-binding-site-leucine-rich-repeat (NLR) receptor in the ‘Baltico’ and SQ genomes. In total there were 381 NLRs in SQ and 331 and 366 NLRs in ‘Baltico’ for Hap1 and Hap2, respectively (Supplementary Data Table S8). We suspected that the lower number of NLR genes could be caused by the haplotype-aware assembly or individual differences [41]. Among the six canonical classes of NLRs, we found that CC-NBARC-LRR occupied the largest proportion, from 65.88 to 70.09%, followed by NBARC-LRR, from 15.41 to 18.90%, in these three genomes. We also explored the distribution patterns of the six canonical classes of NLRs in the genomes. The distribution pattern was comparable in different haplotypes, but small differences could be detected. For example in nearly 4 Mb of Hap1Chr2 and Hap2Chr1, and in nearly 2.5 Mb of Hap1Chr3 and Hap2Chr3, the classes of NLR were different in these regions (Supplementary Data Fig. S13).

### Correlation between genome architectures and gene expression

**Figure 3:**
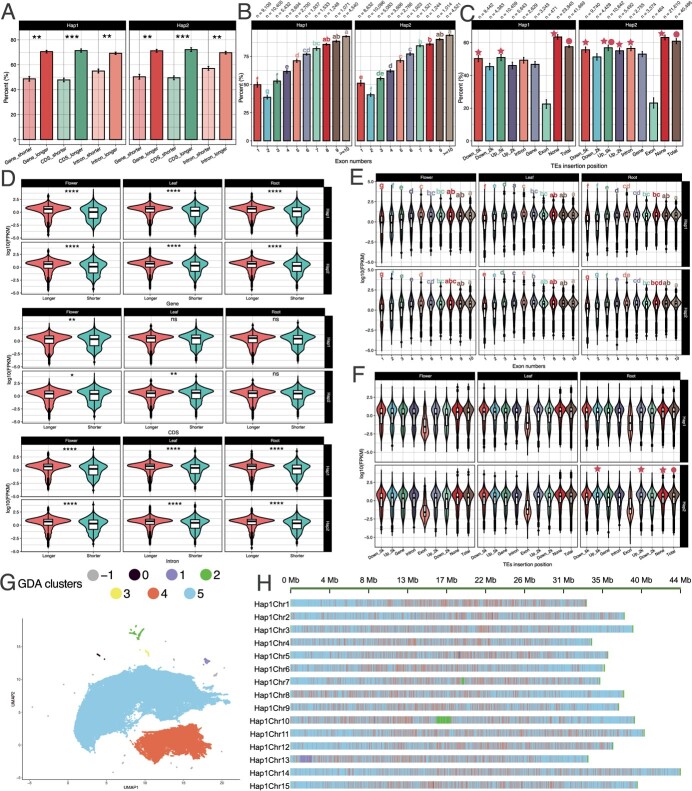
Relationship between gene expression and genome architecture. **A**, **D** Expression ratio (**A**) and FPKM (**D**) of different lengths of CDSs, introns and genes among the two gap-free haplotype-resolved genomes. *P ≤ 0.05; **P ≤ 0.01, ***P ≤ 0.001; ****P ≤ 0.0001; ns, no significant difference. **B**, **E** Expression ratio (**B**) and FPKM (**E**) of different lengths of CDS, introns, and genes. Letters a–g refer to results of significant difference analysis. **C**, **F** Different expression ratio (**C**) and FPKM (**F**) of genes with specific intact TE insertions. The orbicular indicates the group exhibiting no significant difference from Total genes; the pentagram refers to the particular group that exhibited no significant difference from the None groups. ‘Total’ refers to total annotated genes in the genomes; ‘None’ means the genes did not have correlation with TE insertion; ‘n’ refers to the total number of the specific group. **G** Hap1 and Hap2 separated into seven clusters through GDA. **H** Location of the seven clusters classified by GDA among chromosomes of the Hap1.

Previous studies have shown that the lengths of exon, gene, and intron, and TE insertions could affect gene expression levels [20, 42]. We therefore investigated whether these factors could influence expression levels in *D. caryophyllus* based on our gap-free and well-annotated ‘Baltico’ genomes. For the ratio of expressed genes to unexpressed genes, we found that genes with longer lengths of CDS, intron, and gene tended to be expressed in different tissues (Fig. 3A, Supplementary Data Fig. S14A), and genes with longer length (whatever the gene, CDS, or intron) exhibited significantly higher expression ratios than shorter genes.

Genes with different exon numbers exhibited different expression ratios. We found a trend showing that the expression ratio increases with the number of exons for the genes in different tissues (Fig. 3B, Supplementary Data Fig. S14B). Furthermore, we found that genes with two exons exhibited the lowest expression ratio compared with genes with different exon numbers. As there were different expression ratios among different exon numbers, we further checked the possible major function of those genes with a specific number of exons by performing KEGG enrichment analysis. The KEGG enrichment analysis showed that the main classes of ‘BRITE hierarchies’ and ‘metabolism’ were enriched in all groups with different exon numbers. ‘Organismal systems’ was shared among the genes containing 7, 8 and >10 exons; ‘environmental information processing’ was shared between genes containing 1, 2, 3 and >10 exons; ‘genetic information processing’ was present in all groups except for the genes containing seven exons (Supplementary Data Table S9), indicating the preference of gene function in genes with different exon numbers.

We identified a total of 70 563 and 76 690 intact TEs in Hap1 and Hap2, respectively (Supplementary Data Table S10). It seems that in *D. caryophyllus* TEs were more likely to have inserted into a region flanking the gene and these insertions tended to be in the upstream region. Of those TEs that did insert into gene loci, most inserted in introns (Fig. 3C). We found that the TE insertions also correlated with gene expression ratios. Genes correlated with TE insertions had significantly lower expression ratios compared with genes uncorrelated to TE insertion. While TEs inserted into the upstream 5 kb and intron region in Hap2, there were no significant differences compared with genes uncorrelated with TE insertions. Genes with TE insertions located in exons had the lowest expression ratios in both haplotypes in all tissues (Supplementary Data Fig. S14C), and exhibited significant differences when compared with all other insertion types or non-insertion types (Fig. 3C). These results demonstrated that the lengths of CDS, intron, and gene, and the exon numbers of gene and specific TE insertions correlated with the expression ratio.

We further checked the expression levels of the expressed genes whose expression ratio may be affected by different genome architectures. In different tissues, we found that Hap1 in leaf and both Hap1 and Hap2 in root exhibited no significant differences in expression level when compared with different lengths of CDS, while other tissues or haplotypes all showed significant differences in expression levels when comparing longer and shorter genes (Fig. 3D). The general patterns showed that longer genes, CDSs, and introns tend to have higher expression levels than shorter genes. There was a clear pattern of genes with more exons being expressed at higher levels. This was especially the case for genes with exon numbers greater than five compared with genes with exon numbers less than two, which showed significantly higher expression levels in all tissues and haplotypes (Fig. 3E). TEs can also play an important role in the direct or indirect regulation of gene expression. Significantly lower expression levels were observed in both haplotypes in different tissues when there were TE insertions in the exon regions compared with genes without TE insertions and total genes (Fig. 3F). In different tissues of Hap1, we found that the expression levels of genes with TE insertions were significantly lower than the expression levels of genes devoid of TE insertions and the total. In Hap2, we found that the expression levels of genes with TE insertions were significantly lower than those of genes devoid of TE insertions and the total; however, there were differences for the root, where the TE insertions in the upstream 5-kb regions and upstream 2-kb regions were not significantly different compared with the total. We suspect that the haplotypes might be affected differently by the TEs, and this further demonstrates the divergence between the haplotypes.

As TE insertion correlated to lower expression ratio and level, we were curious about whether TE insertion was correlated to functional preference. Through KEGG enrichment analysis, we could significantly enrich several KEGG terms in the genes with TE insertions in the gene and intron region (Supplementary Data Table S11). These KEGG terms mainly correlated with metabolism processes. We also found that the term ‘00940 phenylpropanoid biosynthesis’ for the conversion of anthocyanidins to anthocyanins was enriched [43]. We found that only three KEGG terms were significantly enriched in the TE non-insertion gene set (Supplementary Data Table S11); for example, the term ‘00194 photosynthesis proteins’ was enriched, which may suggest that photosynthesis is not suppressed. The GO annotation results also suggested that TE insertions may have function preference. The genes annotated with terms of ‘catalytic activity’ and ‘binding’ from the main class of ‘molecular function’ (MF), and ‘metabolic process’, ‘cellular process’, and ‘response to stimulus’ from the main class of ‘biological process’ (BP) have significantly different percentages among different TE insertion situations (Supplementary Data Fig. S15). For genes annotated with the GO term ‘catalytic activity’, TEs tend to insert more into gene and intron regions and less into exon and downstream 2-kb regions. For genes annotated with the GO term ‘binding’, TEs tended to insert into exon regions; for genes annotated with the GO terms ‘metabolic process’ and ‘cellular process’, TEs were less likely to insert into exon regions. For genes annotated with the GO term ‘response to stimulus’, TEs were less likely to insert into the gene and intron region and more likely to insert into the exon region.

### Genome decomposition analysis

The gap-free genome provided a great opportunity to study the genome architectures. Through genome decomposition analysis (GDA), we divided the T2T haplotypes into seven clusters based on the specific characteristic of sequences under the non-overlapping window size of 10 kb. The proportion of different clusters in the genome was 0.35, 0.01, 0.27, 0.42, 0.11, 22.75, and 76.00% from clusters −1 to 5 (Fig. 3G), respectively. A total of 27 features were used to perform cluster analysis and 19 features were used to describe the characteristics of regions that could not be clustered (−1); 16, 18, 21, 11, 20 and 21 features were used to describe the characteristic of clusters from 0 to 5 (Supplementary Data Table S12).

Clusters 1 and 2 were classified by the high ratio of tandem repeat regions when compared with other clusters. The main difference between cluster 1 and cluster 2 was that cluster 2 contained more Gypsy and other TEs. We could only detect the centromere candidate region in cluster 2 in Chr10 and cluster 1 in Chr13 with the presence of continuous long blocks.

Cluster 0 shared several characteristics with cluster 1, but has the lower AT skew; thus there were proportionately fewer in the whole genome. Cluster 3 was uniquely characterized by its telomere sequences, mainly existing in the head and the end of each chromosome, which could be identified as telomere regions. Clusters 4 and 5 account for most of the genome (98.75%), indicating that the two clusters represent the main structural characteristics. Cluster 4 had a higher CpG island percentage, fewer complex repeats and inverted repeats, but more repeat-rich regions including retrotransposon proteins, putative retrotransposons and TEs. Cluster 5 contained the fewest TEs and the highest numbers of genes and exons, the longest gene length and highest RNA sequence coverage (Supplementary Data Table S12). In terms of distribution on the chromosomes, cluster 5 has more continuous long block regions, but cluster 4 tends to insert into the long blocks of cluster 5 regions (Fig. 3H, Supplementary Data Fig. S16).

The telomeres of Hap1Chr9, Hap2Chr9, and Hap2Chr7 were in cluster 2. We speculate that the shorter telomere repeat lengths and other significant features may contribute to the cluster results (Fig. 3H, Supplementary Data Fig. S16, Supplementary Data Tables S5 and S12). For clusters 4 and 5, irrespective of the different haplotypes or chromosomes, the ratio was stable (Supplementary Data Fig. S17). Other clusters exhibited different proportions among the different haplotypes. For example, Hap2 contained a greater cluster 1 and 3 content than Hap1, particularly in Chr13. Our GDA gave a more visual correlation between the TE contents and genes, the TE-rich regions were very fragmented and inserted into gene regions.

### Comparative analysis between gap-free haplotypes

We identified the syntenic regions and structure variations between the two gap-free haplotypes. The percentage of syntenic regions between Hap1 and Hap2 in the different chromosomes ranged from 55.88 to 99.99% and 57.13 to 99.93%, respectively (Supplementary Data Table S13). A total of 54 inversions and 973 translocations were identified (Fig. 2A, Supplementary Data Table S13). The most divergent chromosomes were Chr2, Chr3, Chr4, Chr8, and Chr11, these five chromosomes had high percentages of inversions (44) and translocations (79) with respect to total variations. We identified a total of 584 486 SNPs and 115 701 indels (57 508 insertions and 58 193 deletions) between Hap1 and Hap2. Among them, 88 689 of these SNPs and indels were distributed in the exon regions, and 34 608 of these SNPs and indels caused missense mutations. Furthermore, we also investigated whether these indels and highly divergent regions (HDRs) were mediated by intact TEs (Supplementary Data Table S14). There were a total of 30 878 indels and HDRs whose length was >40 bp, and we found that 7311 structural variations may be mediated by TE insertions, accounting for 23.67% of the total number.

**Figure 4:**
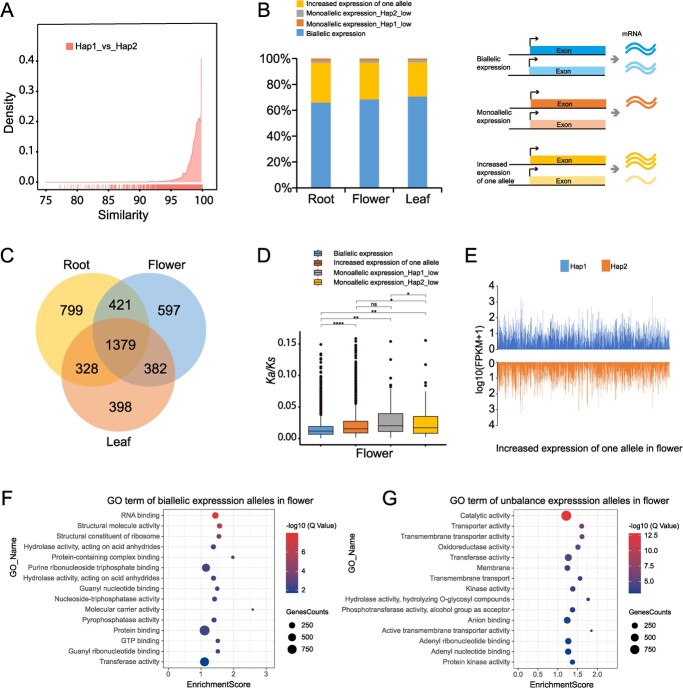
Allele-specific expression characteristics. **A** Similarity of allele CDS between two haplotypes. **B** Statistics on the number of different types of ASE in flowers, roots, and leaves. **C** Venn diagram indicating the number of ASEs in three tissues. (D) *K*_a_/*K*_s_ values for different classes of ASE in flowers. Dots represent outliers. Boxes represent 25–75% of the value. The upper and lower horizontal lines represent the range with 1.5 interquartile range. **E** Distribution of ASEs (class of ‘increased expression of one allele’) in two haplotypes in flowers. Expressions are presented as log_10_(FPKM + 1). *P ≤ 0.05; **P ≤ 0.01; ***P ≤ 0.001; ****P ≤ 0.0001 (*t*-test). **F** GO enrichment analysis of class of biallelic expression in flower. **G** GO enrichment analysis of class of unbalanced expression alleles in flower.

Subsequently, we identified 10 256 alleles that contain at least one SNP variation (20 512 genes, accounting for 24.97% of all annotated genes) between the two haplotypes, alongside 16 036 ‘single alleles’ with identical CDSs between these haplotypes. The CDS similarity of most alleles ranged from 95 to 99% (Fig. 4A). Notably, the similarity of genes between the two haplotypes was comparable to that observed between the two cultivars (Supplementary Data Fig. S18).

To gain insights into ASE of 10 256 alleles, we conducted an analysis using transcriptome data from the blooming flowers, roots, and young leaves of ‘Baltico’. In the three tissues, we found that about 31.55, 33.94, and 29.28% of the expressed genes showed ASE in flowers, roots, and leaves, respectively. Among them, 2907, 2779, and 2487 alleles showed unbalanced expression in roots, flowers, and leaves, respectively (Supplementary Data Table S15). We found that there were more biallelic expression genes in leaves (70.72%) than in flowers (68.45%) and roots (66.06%), and a greater frequency of increased expression of one allele in roots (30.76%) than in other tissues (28.42 and 26.58% respectively in flowers and leaves) (Fig. 4B, Supplementary Data Table S15). Among the total of 4284 expressed alleles in each of the three tissues, 1379 exhibited significantly different expression (Fig. 4C). There were 799, 398, and 597 alleles showing unbalanced expression in roots, leaves, and flowers, respectively. The *K*_a_/*K*_s_ values of monoallelic expression were higher than for alleles of biallelic expression in flower (Fig. 4D), leaf, and root (Supplementary Data Fig. S19). The biallelic expression alleles had significantly lower *K*_a_*/K*_s_ values than any other expression type in the three tissues, indicating that most of the biallelic expression alleles were evolutionarily conserved. We noticed that the increased allele expression in three tissues of both haplotypes showed no bias (Fig. 4E, Supplementary Data Fig. S20). GO enrichment analysis revealed that the biallelic expression alleles of flowers were primarily enriched in terms related to ‘RNA binding’, ‘structural molecule activity’, and ‘structural constituent of ribosome’ (Fig. 4F). For the alleles showing unbalanced expression alleles in flowers, the terms ‘catalytic activity’, ‘transporter activity’, and ‘transmembrane transporter activity’ were significantly enriched (Fig. 4G). In addition, as in flowers, the alleles showing biallelic expression and the alleles showing unbalanced expression in roots and leaves were enriched in similar GO terms, which suggests that there was no significant tissue specificity of differentially expressed alleles (Supplementary Data Fig. S21).

To check whether ASE might correlate with TE insertions, we further detected 1098, 964, and 1151 ASEs with specific TE insertions in flower, leaf, and root, accounting for 38–39% of the total ASE numbers (Table 2, Supplementary Data Tables S16 and S17). The ASEs in different tissues between the haplotypes may play important roles in the formation of carnation traits. For example, one ASE annotated with ‘UDP-D-xylose’ was reported to be involved in the biosynthesis of a branched-chain sugar [44] and contained a specific DNA/DTH insertion in the upstream 5-kb region in Hap1 (Supplementary Data Fig. S22A), the expression of which was significantly lower in both haplotypes of the three tissues. Consistent with the general pattern that TE insertion correlated with lower expression level, alleles with specific TE insertion showed significantly lower expression than alleles without TE insertion.

**Table 2:** Numbers of ASEs and ASEs with specific TE insertions between alleles among different tissues.

	Flower	Leaf	Root
Total ASE number	2779	2487	2907
Specific TE insertions in ASE	1098 (39.51%)	964 (38.76%)	1151 (39.59%)
Specific TE insertions in ASE in Hap1	436	370	459
Specific TE insertions in ASE in Hap2	662	594	692

## Discussion

### The telomere-to-telomere ‘Baltico’ genomes provide a new insight into the genome structure of *D. caryophyllus*

The previous lack of an accurate and gap-free genome presented a significant barrier in tracking and understanding repeat structure, function, and variation in large complex repeats [9] such as found in the centromere and telomere regions. In this study, we assembled and annotated an accurate, continuous, and complete gap-free *D. caryophyllus* genome based on high-depth long-read sequencing data and state-of-the art assembly methods. This finished genome provided an opportunity to analyze the genome-scale repeat content and identify genome architecture.

The centromeric region is important for faithful chromosomal segregation in mitosis and meiosis, and deletion of the centromere or mutation of critical kinetochore proteins results in chromosome loss [45, 46]. Generally, centromeres in most higher eukaryotic organisms are composed of long arrays of satellite DNA [47, 48], which can be identified by the abundance of a repeat monomer (often >10 000 copies per chromosome) [49]. The centromeric region size can range from ~500 kb to several megabases [50, 51] and the length of repeat monomers is ~180 bp, also could found in a broader length in animals [49].

In some plants and animals, a single chromosome, or even the entire chromosome complement, lacks HOR arrays [46, 52–54]. For example, there are five centromeres in potato, in which the HORs could not be identified; six different repeat monomers were identified and four of the centromeric repeats were amplified by the retrotransposon-related sequences [54], which provides great genetic diversity in the centromere among different species. In the three carnation genomes (two haplotypes of ‘Baltico’ and SQ), we detected only four candidate centromere regions by identifying the HORs in ‘Baltico’. Bioinformatic analysis of our gap-free genome led to the conclusion that the carnation’s centromere has specific characteristics that cannot be identified by HORs alone.

The telomere regions cap the ends of eukaryotic chromosomes to protect them from deterioration and prevent a DNA damage response [55], and consist of a tandem repeat [56]. The difference in telomere length among plants is correlated with certain phenotypes [57, 58]. For example, telomere length variation may be associated with flowering time [59]. Our gap-free genome provided a valuable resource for analyzing flowering time correlated with telomere region length in the Caryophyllales.

### Correlation between gene expression, gene structure, and transposable element insertion

More and more studies are focusing on ASE, such as on the types of ASE, the causes of ASE and the regulatory mechanisms involved in the formation of important traits [60, 61]. ASE has been reported to affect individual traits such as color [20] and resistance [22, 24]. We found a large amount of ASE (29.28–33.94%) in flower, leaf, and root. These ASEs were divided into four different types and enriched in different terms, suggesting that different classes of ASEs may be involved in different regulatory pathways.

The exon numbers and gene and intron lengths have been reported to affect gene expression levels, showing that genes with longer intron lengths, more exons, or with TE insertions are more likely to exhibit higher expression levels than genes with shorter lengths or genes without TE insertions [42]. Researchers found that high levels of expression tended to be associated with shorter mRNA lengths [62]. However, in our case, based on the gap-free and well-annotated genomes, we found that the genes with longer CDSs, introns, and genes would tend to be expressed and at a higher expression level than shorter ones (Fig. 3A and D). Former studies demonstrated that shorter genes correlate with the stimuli [63], and the longer genes are often associated with important biological processes [64, 65].

Our results reveal that the gene expression ratios and expression levels are correlated with TE insertions. Genes without a TE insertion have higher gene expression ratios and levels than genes with TE insertions. It seems that TEs are mainly characterized as repressive elements in *D. caryophyllus* (Fig. 4C and F). In particular, TEs inserted into the exon regions significantly correlate with downregulation or gene silencing. This downregulation process correlated with TEs may be achieved by the specific insertion disrupting the genes normal structure [66]. In our case, we found that specific DNA/DTH insertions in the flanking gene regions correlated to significantly lower expression (Supplementary Data Fig. S22B), indicating that the allele imbalance could be caused by the specific TE insertions.

## Materials and methods

### Plant materials and genome sequencing

The carnation variety ‘Baltico’ (2*n* = 30) used in this study was collected from the experimental field of the Comprehensive Experimental Base of Shenzhen Institute of Agricultural Genomics, Chinese Academy of Agricultural Sciences (Shenzhen, Guangdong, China).

For HiFi data, young leaves were collected to extract genomic DNA using the cetyltrimethylammonium bromide (CTAB) extraction method. Subsequently, a PCR-free SMRT library with an insert size of 15 kb was constructed and sequenced using the PacBio Sequel II platform. For each UL nanopore library, ~8–10 μg of genomic DNA was size-selected (>100 kb) using the SageHLS HMW library system (Sage Science, USA). The DNA was then processed using the Ligation Sequencing 1D Kit (SQK-LSK109, Oxford Nanopore Technologies, UK) following the manufacturer’s instructions. Approximately 800 ng of DNA libraries were constructed and sequenced on a Promethion (Oxford Nanopore Technologies, UK) at the Genome Center of Grandomics (Wuhan, China). For Hi-C data, freshly harvested leaves were lysed, and DpnII endonuclease was used to digest fixed chromatin. The DNA’s 5′ overhangs were recovered using biotin-labeled nucleotides and the resulting blunt ends were ligated together using DNA ligase. Proteins were removed with protease to release the DNA molecules from the crosslinks. The purified DNA was then sheared into fragments ranging from 300 to 600 bp. Finally, libraries were quantified and sequenced using the MGI-2000 platform. RNA sequences were obtained from pooled stems, leaves, and flowers of carnation ‘Baltico’ and used for genome structure annotation; young leaves, flowers, and roots were used for ASE analysis. The extracted RNA was used to construct cDNA libraries, which was sequenced on the Illumina HiSeq X platform to generate 150-bp paired-end reads.

### Genome assembly and evaluation

The first assembled contig of the genome was accomplished by using hifiasm [67] (0.19.2-r560), combined with the HiFi reads, UL reads (>100 000 bp), and Hi-C data. Initial assembly results were further filtered by removing the organellar contigs by comparing the mitochondrion genome, chloroplast genome, and nucleotide collection database (nt). 3D-DNA [68] and JUICER [69] were used to sort and orient the contigs into pseudochromosomes and manual curation was performed with Juicebox Assembly Tools (JBAT). Gaps and missed telomeres were further filled using error-corrected ONT data using TGS-gapcloser [70] and manually checked by blastn [71]. Pilon [72] was used to polish ONT reads. KAT [73] and BUSCO v5.2.2 [74] were used to evaluate the quality of the assembled genome by using the databases ‘eudicots_odb10’ and ‘embryophyta_odb10’. The switch errors were evaluated by calc_switchErr (https://github.com/tangerzhang/calc_switchErr). The QV was evaluated by yak (https://github.com/lh3/yak) using the short sequencing data. Furthermore, the quality of the two haplotypes was assessed using the 72 L genetic maps of the published carnation genome [39] using ALLMAPS (https://github.com/allmaps/allmaps).

Tidk (https://github.com/tolkit/telomeric-identifier) was used to identify the position of the telomere in the T2T assembly. Tandem repeats finder (TRF) [75] with ‘1 1 2 80 5 200 2000 -d -h -l 1’, ModDotPlot (https://github.com/marbl/ModDotPlot) and srf (https://github.com/lh3/srf) were used to identify the candidate centromere location of each chromosome.

### Genome annotation

For protein-coding gene prediction, we used homology, *de novo* and transcriptome prediction. Homologue proteins from eight plant genomes, including *Arabidopsis thaliana*, *Oryza sativa*, *Rosa chinensis*, *Vitis vinifera*, *Carica papaya*, *D. caryophyllus*_draft_r1.0, *Solanum lycopersicum*, and *Beta vulgaris*, and the established carnation genome annotation [19], were selected to align to the ‘Baltico’ genome assembly by exonerate software v2.2.0 and AUGUSTUS v3.3.3. For transcriptome prediction, RNA-seq data from stems, leaves, and flowers were mapped onto the ‘Baltico’ genome using HISAT2 v2.1.0 [76]. In addition, Trinity [77] was used to assemble the RNA-seq data, and the result was used to create several pseudo-unigenes. These pseudo-unigenes were mapped onto the ‘Baltico’ genome and gene structures were predicted by PASA v2.5.2 [78]. For *de novo* prediction, AUGUSTUS v3.3.3 [79], SNAP v2013-02-16 [80], and GlimmerHMM v3.0.4 [81] were used to predict coding regions. Gene model evidence from the above programs was combined by EvidenceModeler [78] to get the final non-redundant set of gene structures. The repeat contents were identified using EDTA v2.1.0 [82]. The NLRs were annotated by the NLR-Annotator [83].

### Analysis between haplotypes

The genomes were aligned by minimap2 [84]; variations were further identified using SYRI [85] and were plotted by GenomeSyn [86], the standard for determining whether the structure variations (SV) (indels and HDRs, >40 bp) may be mediated by intact TE insertions according to the description in Supplementary Data Fig. S23A. Homologous regions and syntenic blocks between two haplotypes of carnation were constructed through the alignment of CDS sequences using MCScanX [87]. Allelic genes were identified based on the following criteria: (i) paired regions must be located on homologous haplotypes within syntenic blocks; (ii) a gene and its best homologous gene on another haplotype should be matched; and (iii) a minimum of one SNP variation (insertion, deletion, and variation) is required within the CDS sequence alignment. Genes meeting these criteria were considered as alleles. When genes within syntenic blocks between the two haplotypes shared identical CDSs, they were designated as a ‘single allele’.

To perform the ASE analysis, blooming flowers, young leaves, and roots were isolated for RNA sequencing with three biological duplicates. The raw RNA reads were trimmed and mapped onto the ‘Baltico’ genome by HISAT2, and reads that uniquely mapped were kept for analysis. The count was obtained by HTSeq [88] with the following parameters: ‘-f bam -r name -t gene -i ID -a 0 -s no -m union’. DESeq2 [89] was used to identify differentially expressed genes (alleles showing unbalanced expression) (*P* < 0.05 and |log2FoldChange| > 1). In addition, the expression of alleles was divided into two classes: biallelic expression genes, in which the expression of alleles does not differ between the two haplotypes; and alleles showing unbalanced expression in which there was differential expression. Alleles in the unbalanced expression category were divided into three classes following classification methods reported previously [61]. The three classes were monoallelic expression with Hap1; monoallelic expression with Hap2; and increased expression of one allele. Among these alleles, the partitioning criteria were set as follows. If the count was less than one in one haplotype and greater than one in the other haplotype, it was considered as monoallelic expression (Hap1 or Hap2). The other unbalanced expression alleles were considered as snowing increased expression of one allele. The number of fragments per kilobase of exon model per million mapped fragments (FPKM) was calculated using StringTie v2.1.6 [90] (parameter -e). GO enrichment was visualized using the hiplot online site (https://hiplot.cn).

### Comparison of expression levels between genes with different features

The expression level was obtained from flower, leaf, and root. Intact TEs annotated by EDTA were used to analyze the correlation with gene expression. The regions where the intact TEs inserted into the 5-kb flanking gene regions, 2-kb flanking gene regions, gene region, exon region, and intron region were considered as TEs affecting candidate genes (details can be found in Supplementary Data Fig. S23B). Each gene whose CDS length, intron length, and total gene length was longer or shorter than the median value was placed in the longer or shorter group, respectively. Each gene that exhibited a value of 0 for the average FPKM (flower, leaf, and root) would be identified as a non-expressed gene. The *t*-test and ANOVA were used to analyze for significant differences and a *P*-value of <0.05 was considered a significant difference between the counterparts.

### Genome decomposition analysis

GDA v1.0 [91] was used to perform the analysis. A window size of 10 kb was used to extract the sequence features using default parameters. We added features containing RNA mapping depth, the repeat contents identified by EDTA, and the genome annotation prediction results, and set the telomeric sequence to ‘*Arabidopsis_thaliana*’. A total of 27 features were used for dimensionality reduction and clustering by Python UMAP [92] and hdbscan [93] libraries. According to the Kolmogorov–Smirnov test, the *P*-value <1e−20 was taken to indicate a significant difference.

## Supplementary Material

Web_Material_uhad244
