# Supplementary material for: The haplotype-resolved telomere-to-telomere carnation (Dianthus caryophyllus) genome reveals the correlation between genome architecture and gene expression
Source: Hortic Res. 2023 Nov 27;11(1):uhad244. doi: 10.1093/hr/uhad244 (PMC10788775; doi:10.1093/hr/uhad244)
Supplement: Web_Material_uhad244 [file web_material_uhad244.zip › Supply_figures_change.pdf]

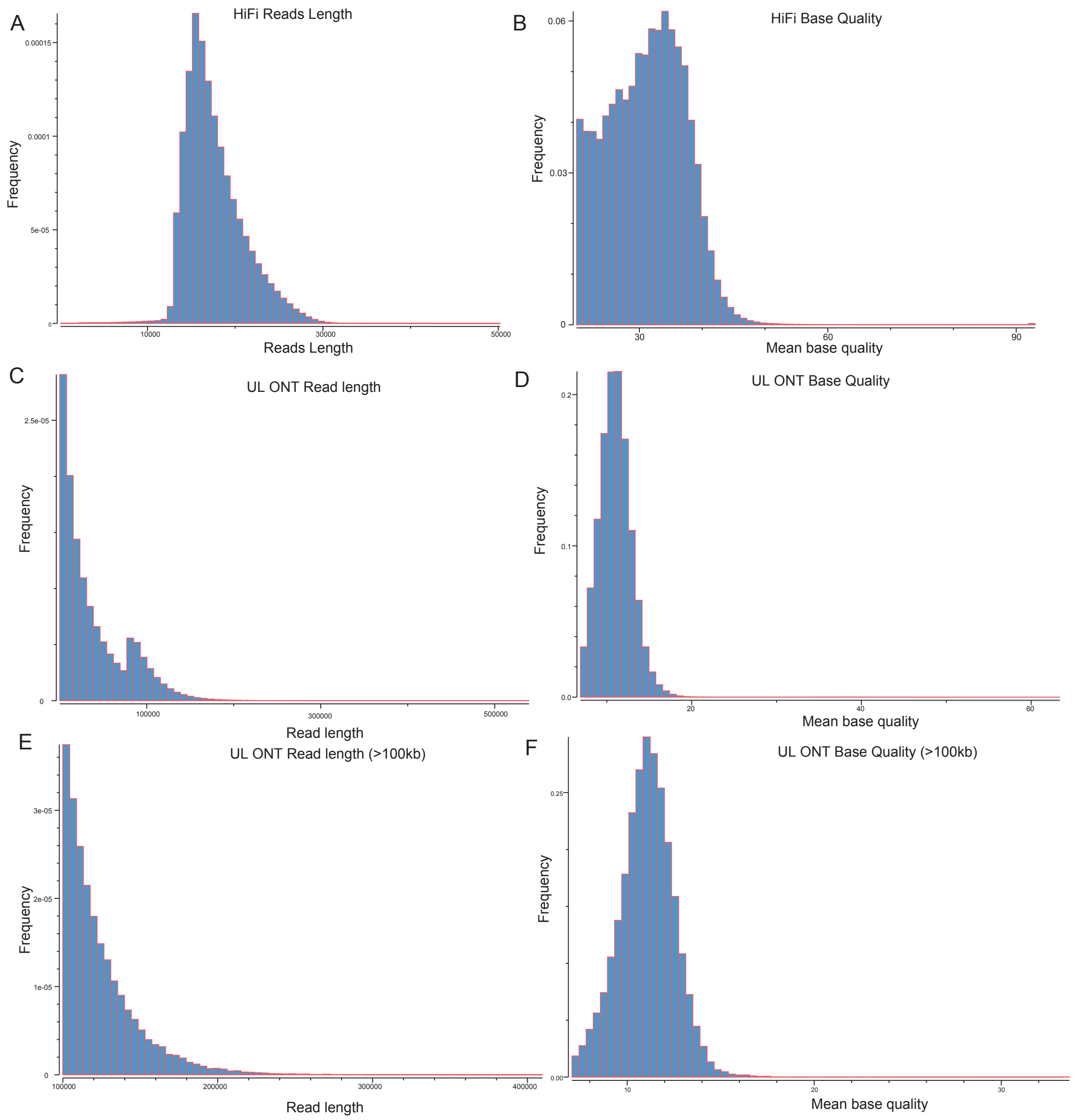

Figure S1. The quality of genome sequencing data. (A) refers to the length of each HiFi reads length; (B) refers to the base quality of HiFi reads; (C) refers to the lengths of each UL ONT reads; (D) refers to the base quality of UL ONT reads; (E) refers to the lengths of each filtered UL ONT (>100 kb) reads; (F) refers to the base quality of UL ONT (>100 kb) reads.

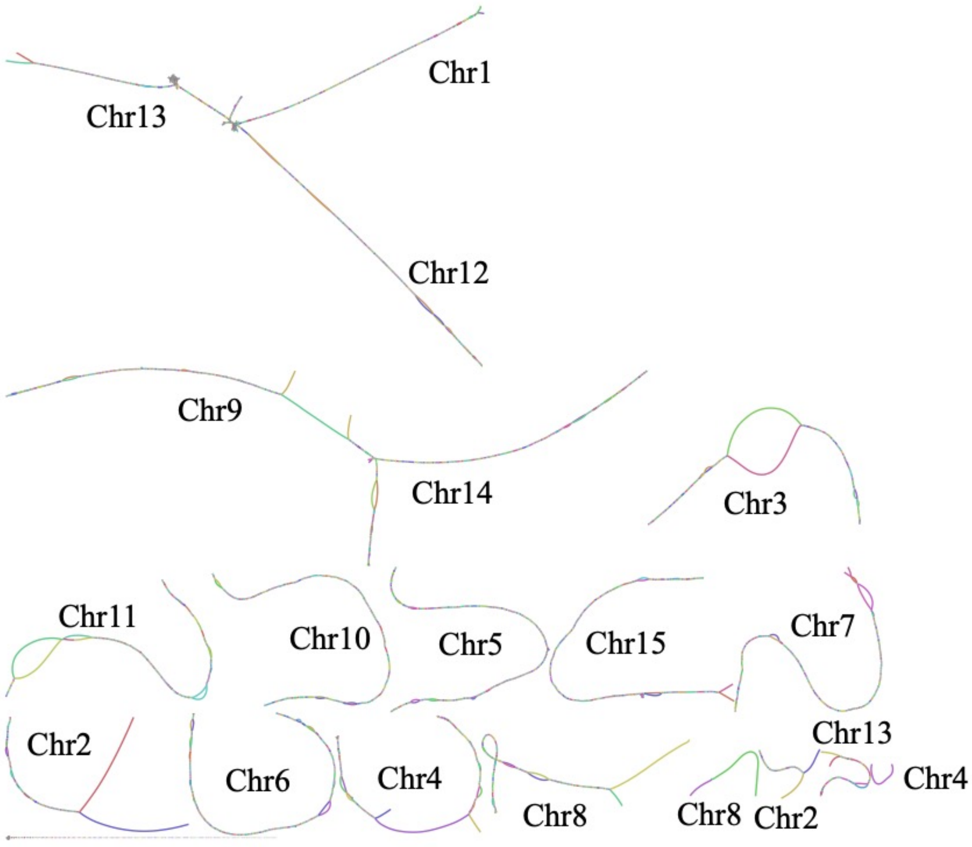

Figure S2. The assembly graph of the *Dianthus caryophyllus* ‘Baltico’.

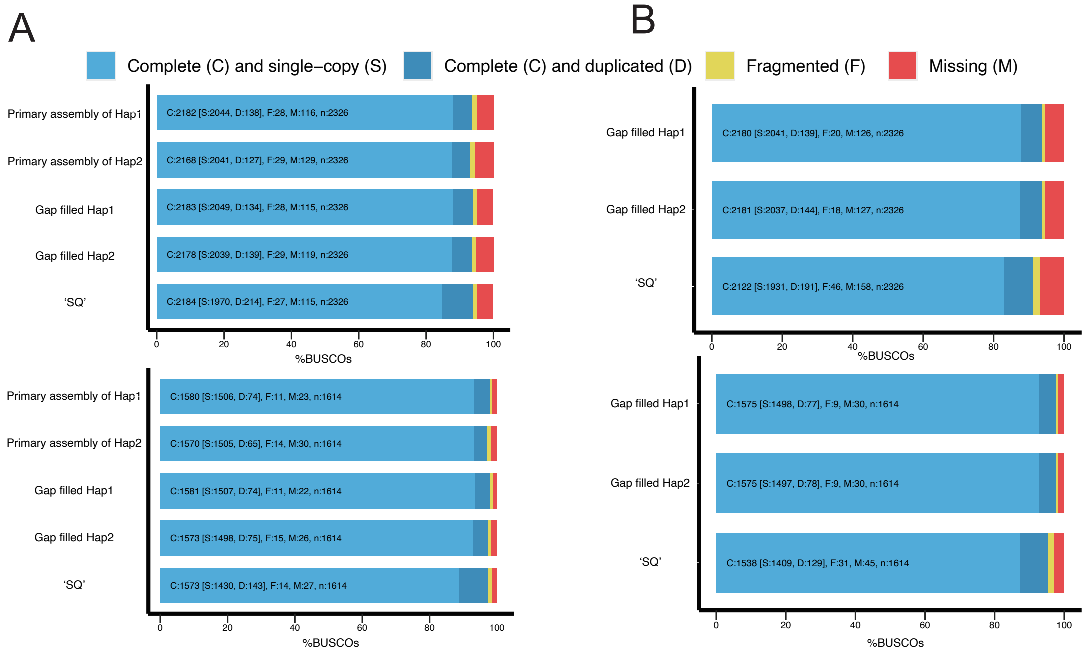

Figure S3. The BUSCO evaluation results of the carnation genomes. (A) the genome assembly evaluation results, the top results were evaluated by “eudicots_odb10”, the bottom results were evaluated by “embryophyta_odb10”; (B) the genome structure prediction evaluation results, the top results were evaluated by “eudicots_odb10”, the bottom results were evaluated by

“embryophyta_odb10”.

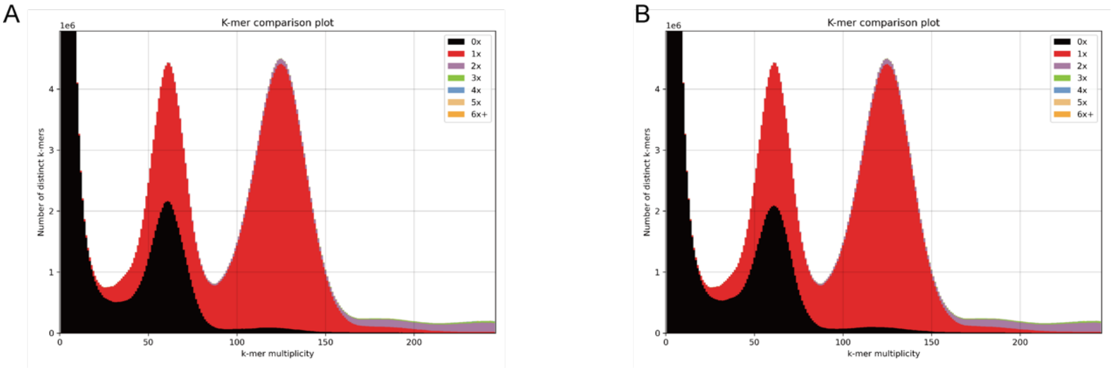

Figure S4. The KAT evaluation kmer spectrum plot to the primary assembly results of *D. caryophyllus* ‘Baltico’. (A) refers to the Hap1 and (B) refers to the Hap2.

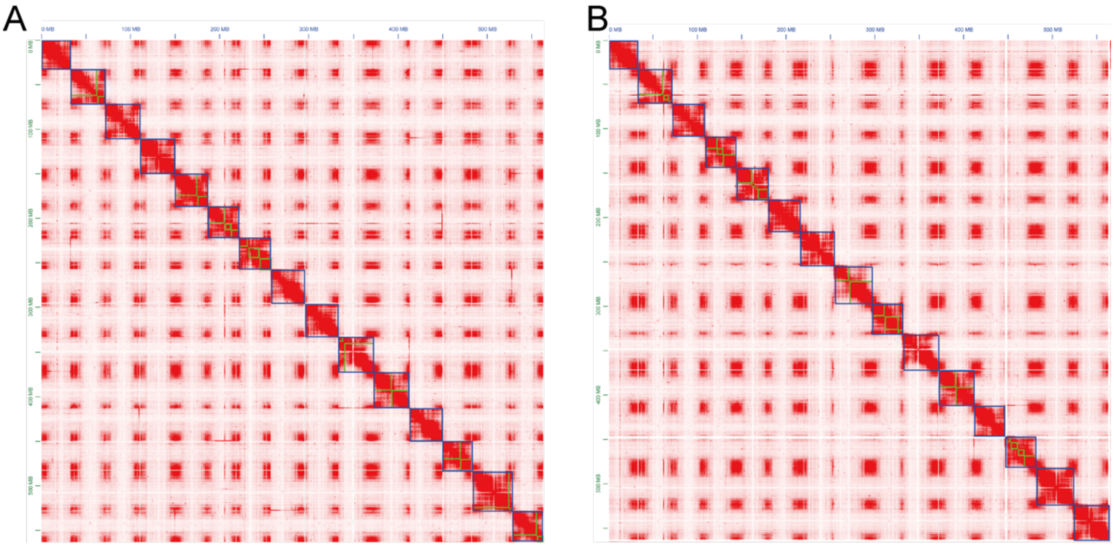

Figure S5. The Hi-C heatmap to the primary assembly results of *D. caryophyllus* ‘Baltico’, the gaps could be directly observed. (A) refers to the Hap1 and (B) refers to the Hap2.

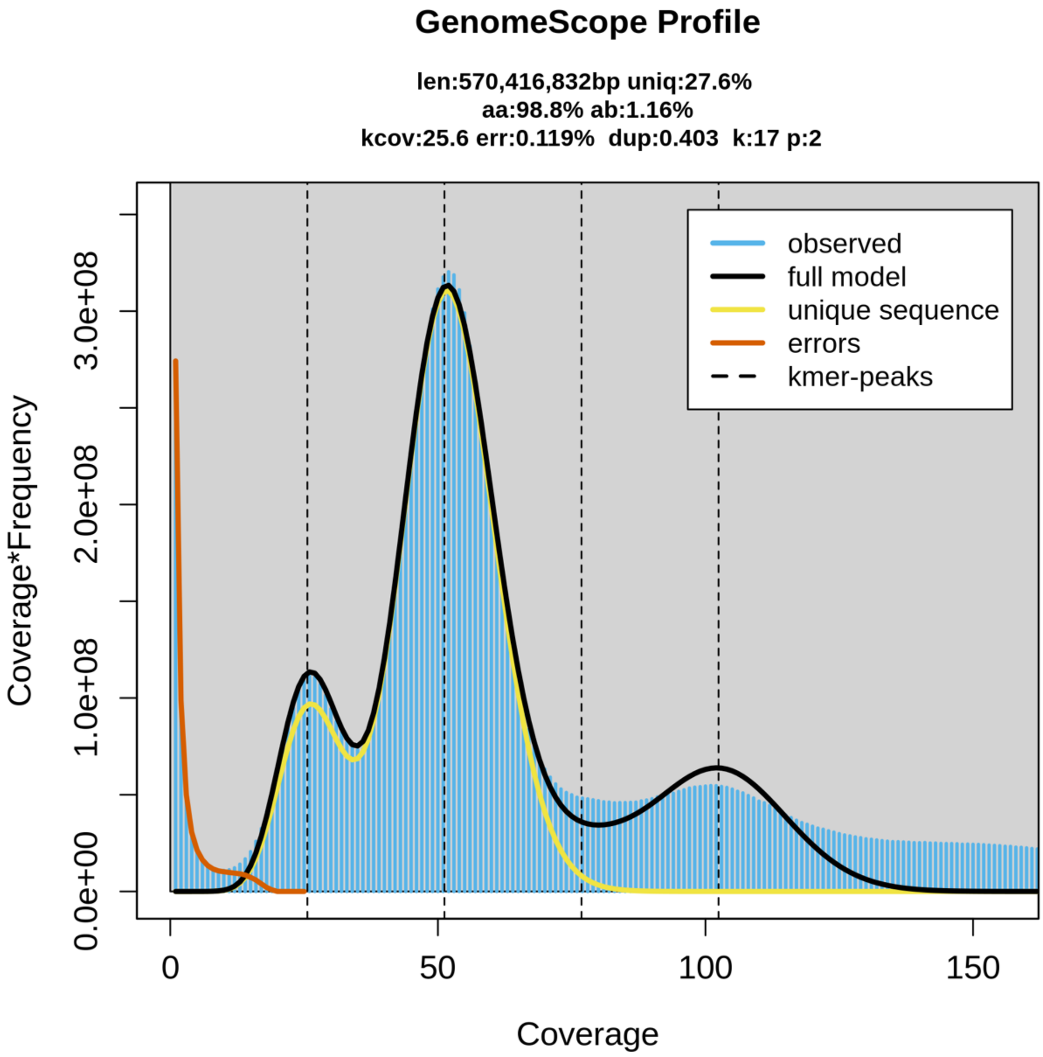

Figure S6. Genome survey results of *D. caryophyllus* ‘Baltico’.

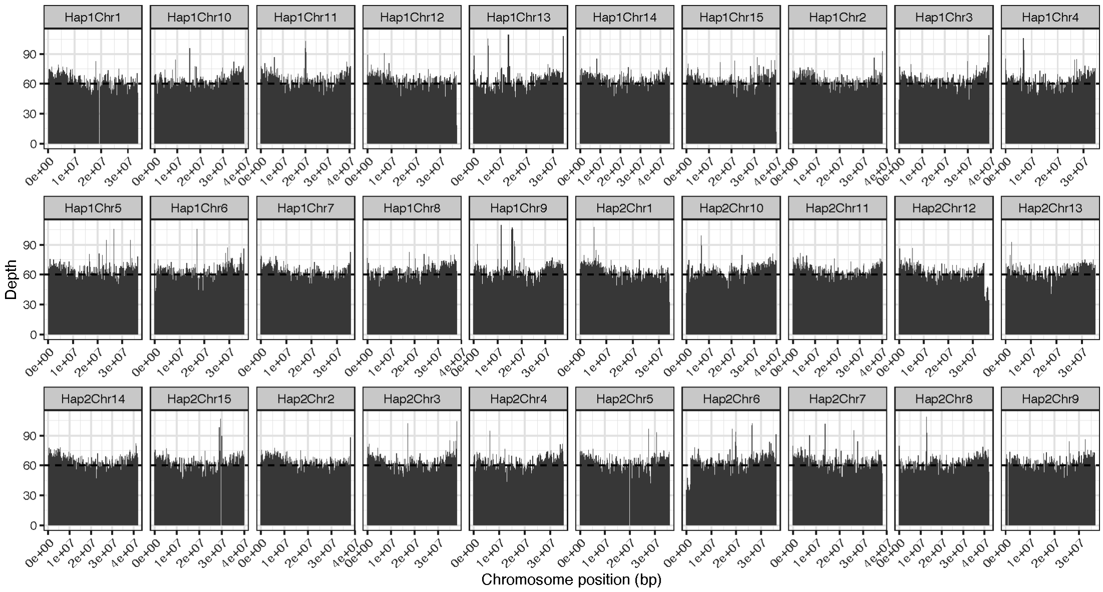

Figure S7. The mapping depth of HiFi data to the assembled T2T genome of *D. caryophyllus* ‘Baltico’.

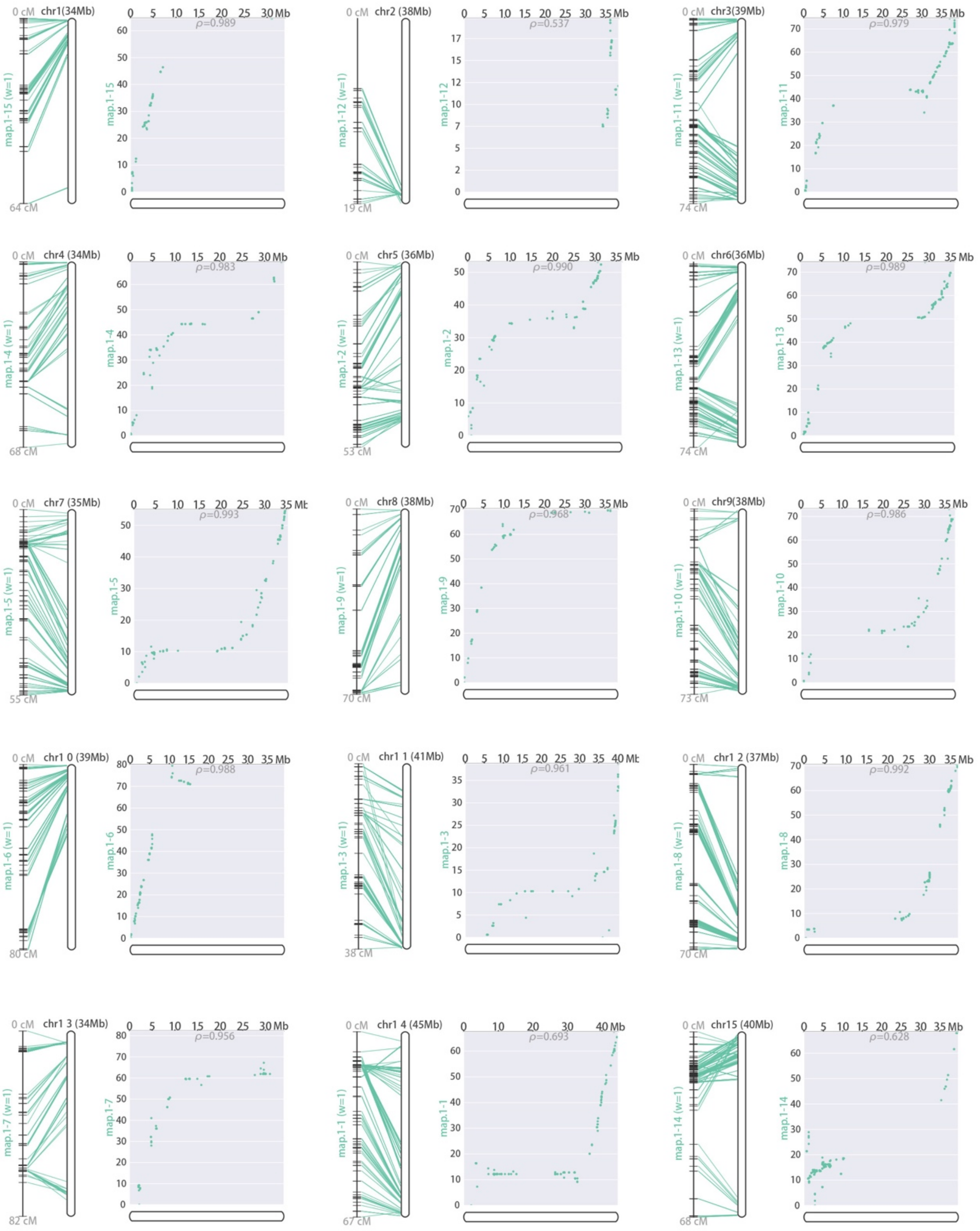

Figure S8. Collinearity between carnation genetic map and Hap1 of ‘Baltico’ psechromosomes. Alignment of the physical sequences of Hap1 of ‘Baltico’ psechromosomes (in Mb) to the ‘72L’ carnation map (in cM).

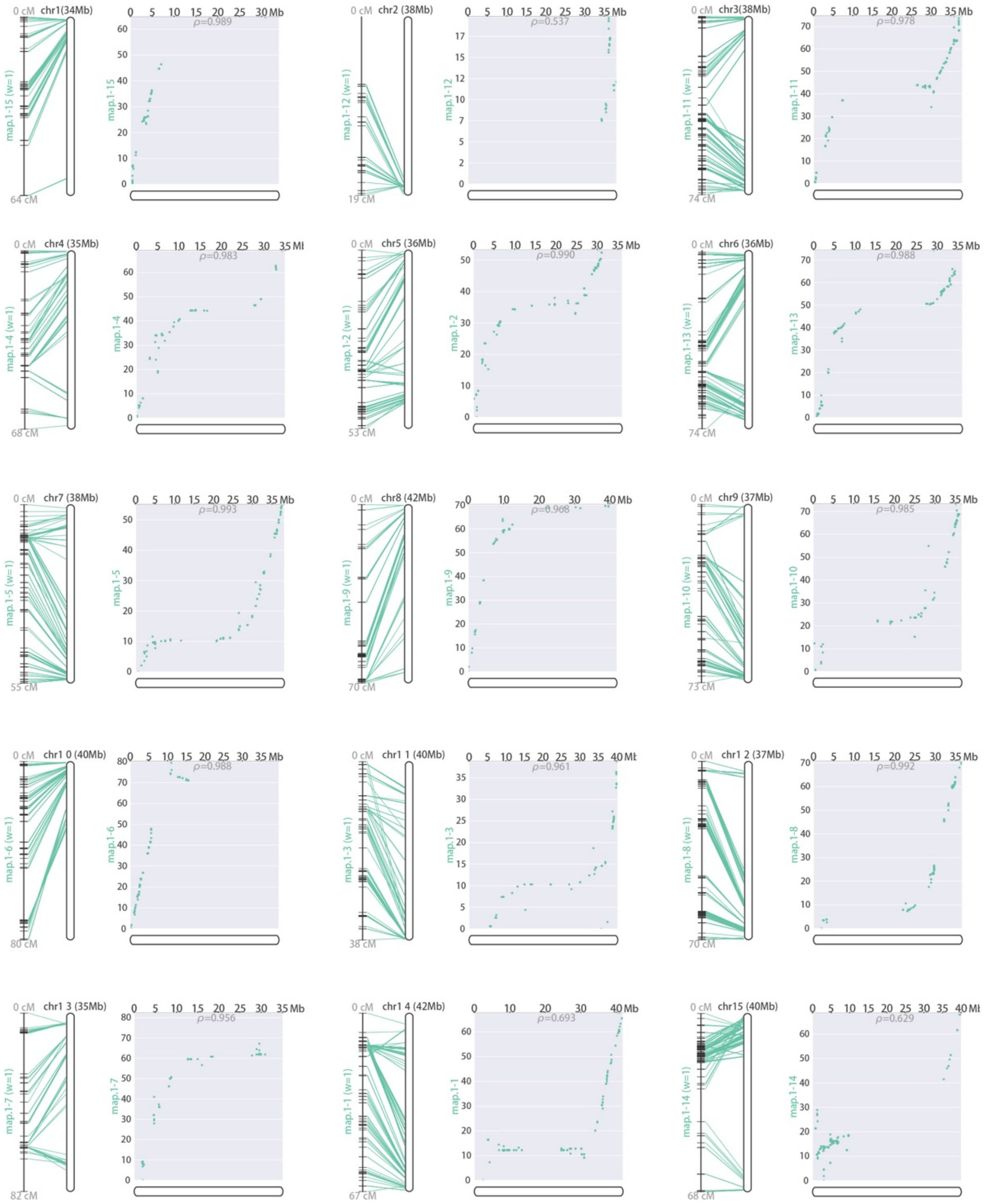

Figure S9. Collinearity between carnation genetic map and Hap2 of ‘Baltico’ psechromosomes. Alignment of the physical sequences of Hap2 of ‘Baltico’ psechromosomes (in Mb) to the ‘72L’ carnation map (in cM).

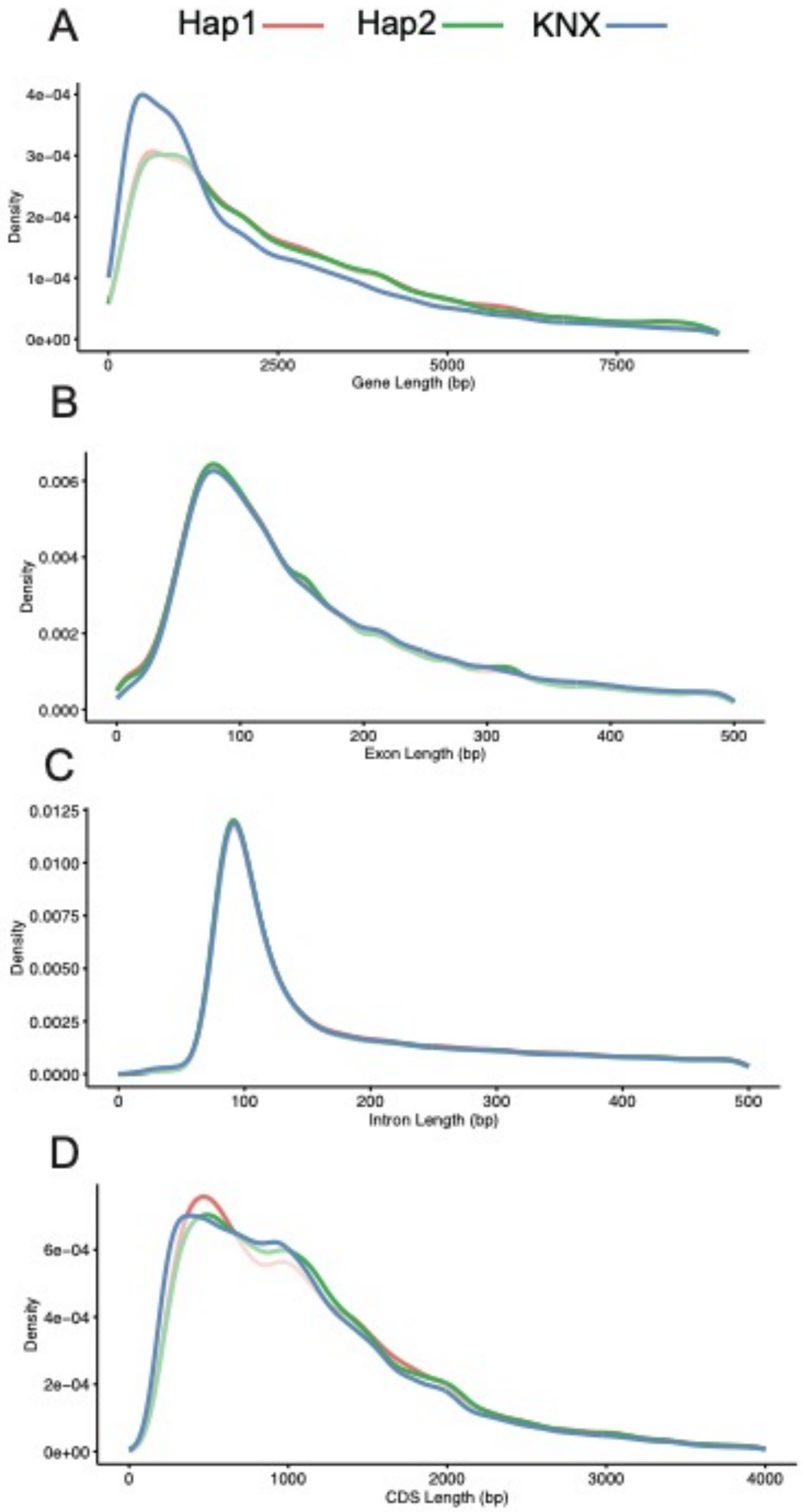

Figure S10. The distribution of length to the (A) gene, (B) exon, (C) intron and (D) CDS. Hap1 and Hap2 represent haplotype of 'Baltico'. 'SQ' represents the genome of 'Scarlet Queen'.

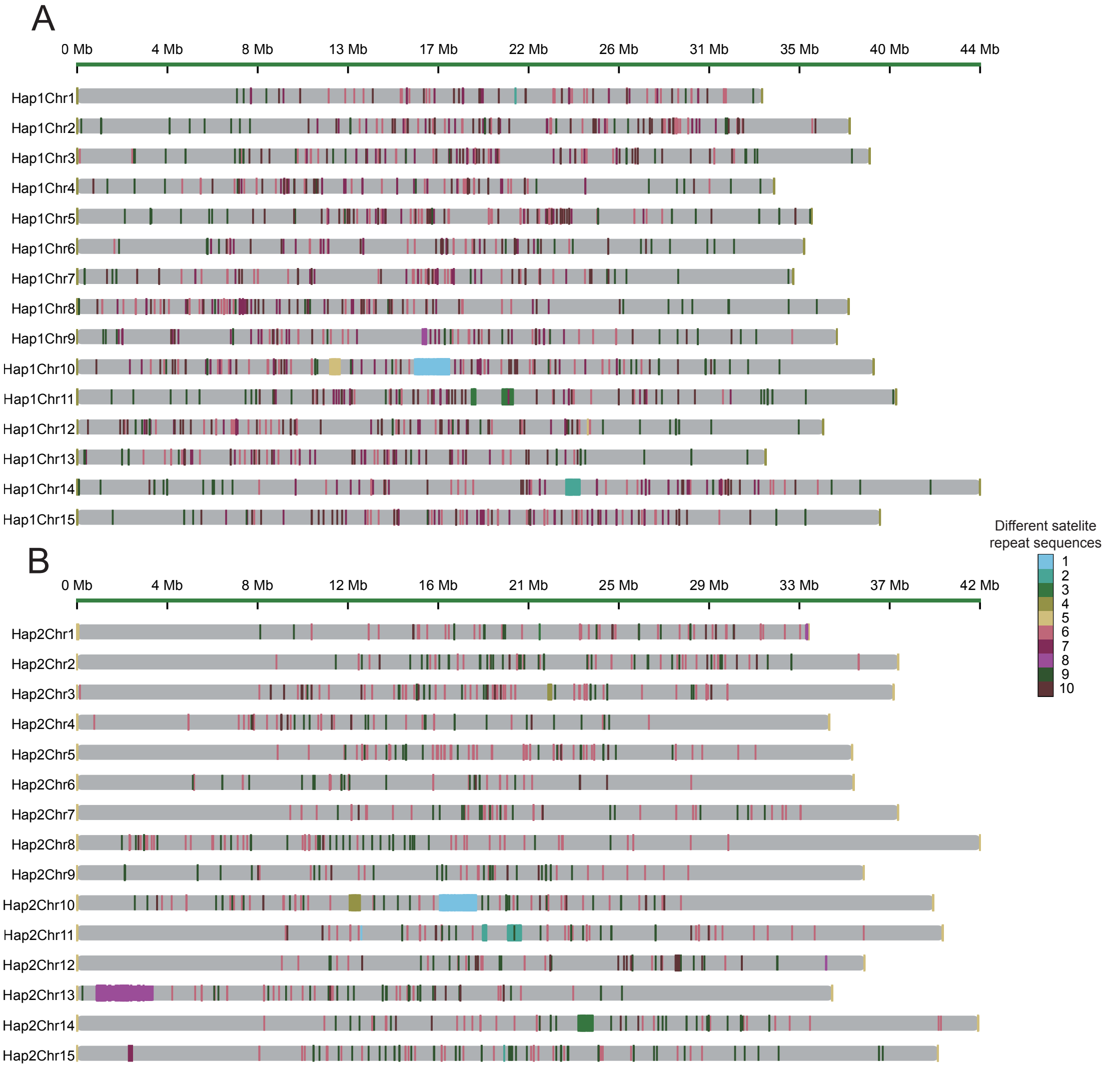

Figure S11. The distribution of satellite repeats sequences identified by the srf among the (A) Hap1 and (B) Hap2, the number from the 1 to 10 represent the most abundant top10 satellite repeat sequences.

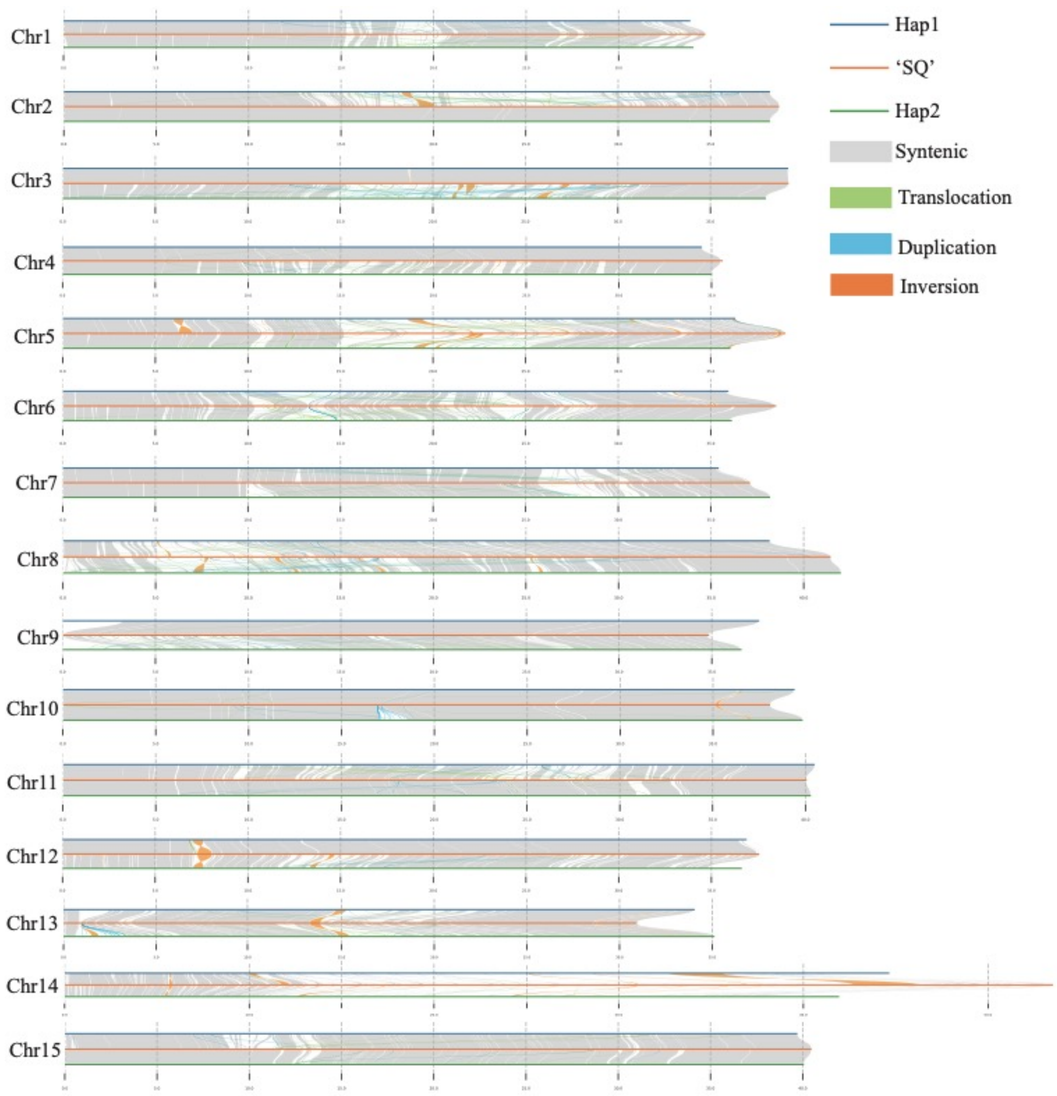

Figure S12. The collinearity analysis between the carnation genome of 'Baltico' and 'SQ'.

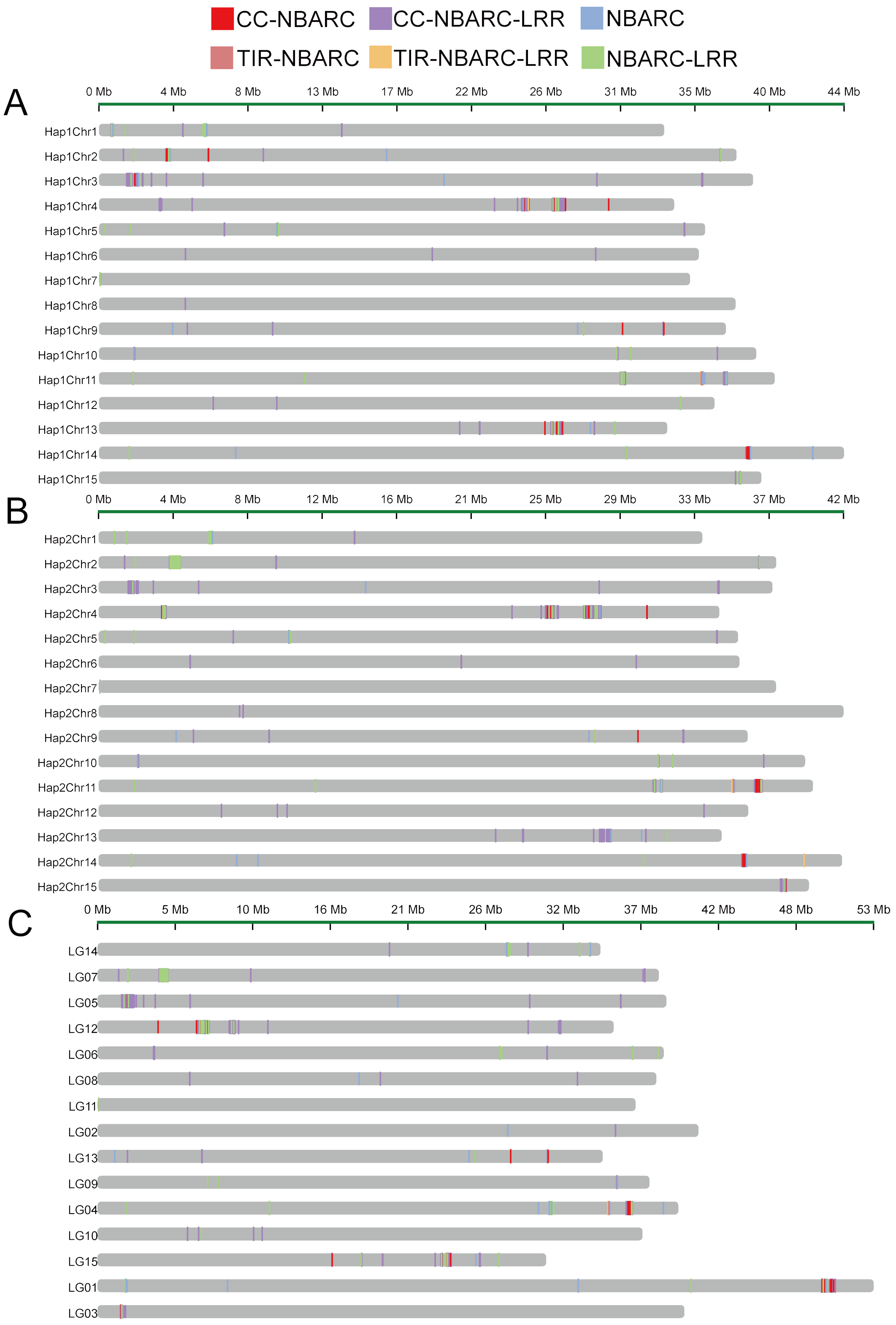

Figure S13. The distribution of NLR in chromosomes among different genomes. (A) and (B) refers to the Hap1 and Hap2 of 'Baltico' respectively, and (C) refers to the 'SQ'.

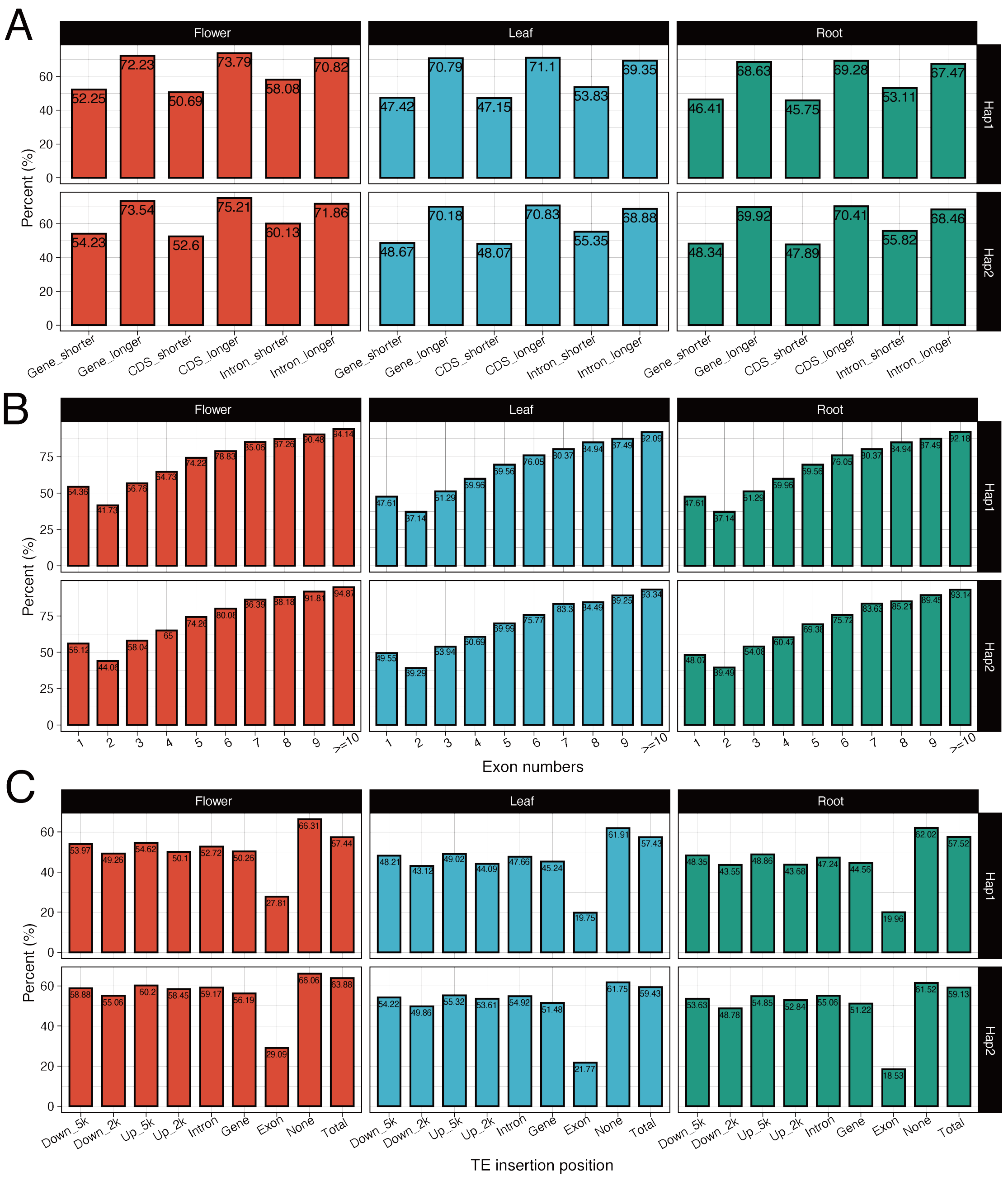

Figure S14. The expression ratio in different tissues of 'Baltico' haplotypes while correlated with different genome architectures. (A) The expression ratio of different length of CDS, intron and gene, (B) the expression ratio of the genes with different exon numbers, (C) the expression ratio of the genes with different TEs insertions type among the two gap-free 'Baltico' haplotypes.

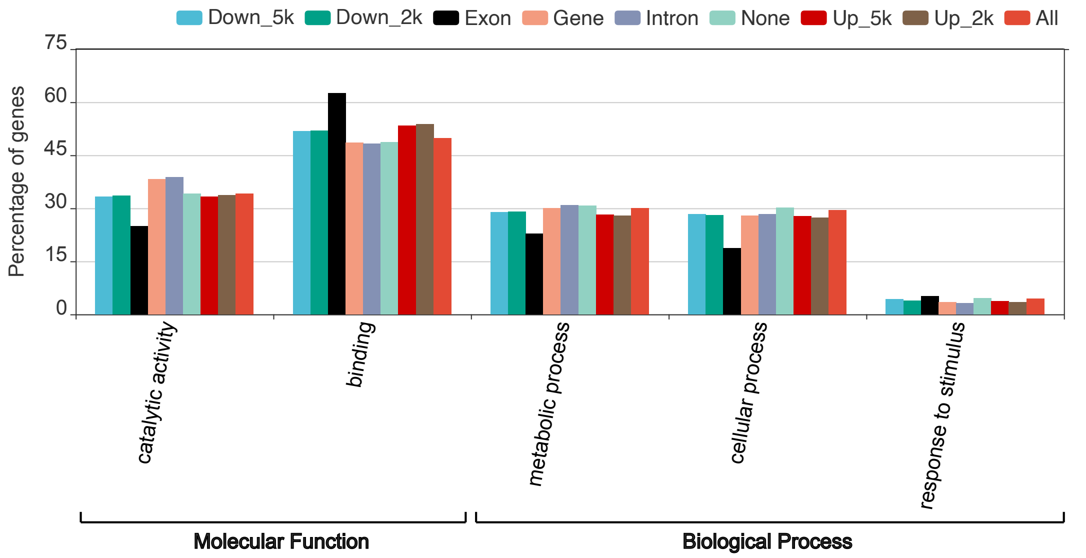

Figure S15. The GO annotation results of the genes with specific TEs insertion. ‘Down_5k’ refers to the TEs inserted into the 5k bp downstream region of the genes; ‘Down_2k’ refers to the TEs inserted into the 2k bp downstream region of the genes; ‘Up_5k’ refers to the TEs inserted into the 5k bp upstream region of the genes; ‘Up_2k’ refers to the TEs inserted into the 2k bp upstream region of the genes; ‘Exon’ refers to the TEs inserted into the exon region; ‘Gene’ refers to the TEs inserted into the gene region; ‘Intron’ refers to the TEs inserted into the intron region; ‘None’ refers to that no TEs inserted into the gene flank regions or inside the gene region. The GO level was set to 2 and only plot the terms exhibited significant difference.

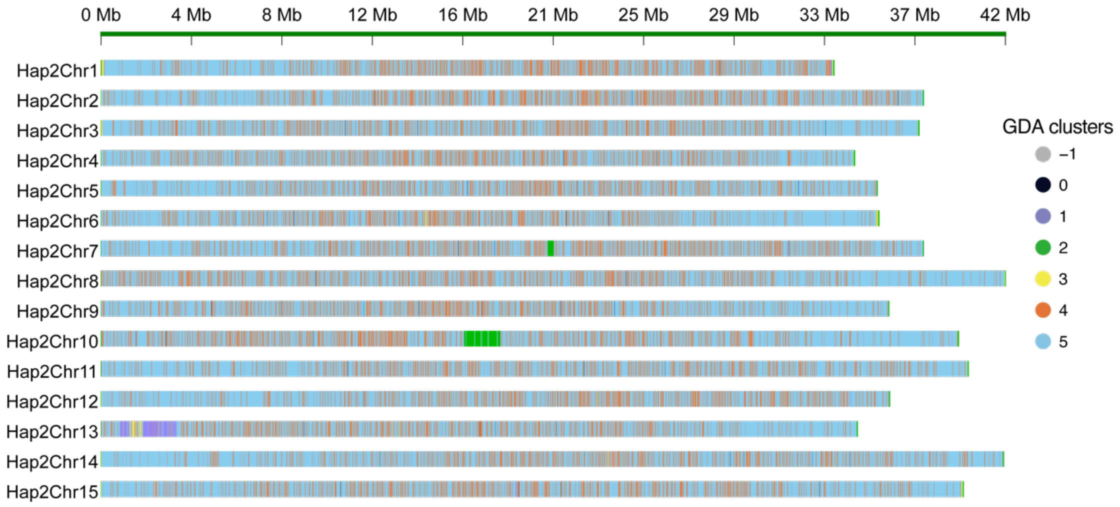

Figure S16. The location of the 7 clusters classified by the GDA among chromosomes of the Hap2.

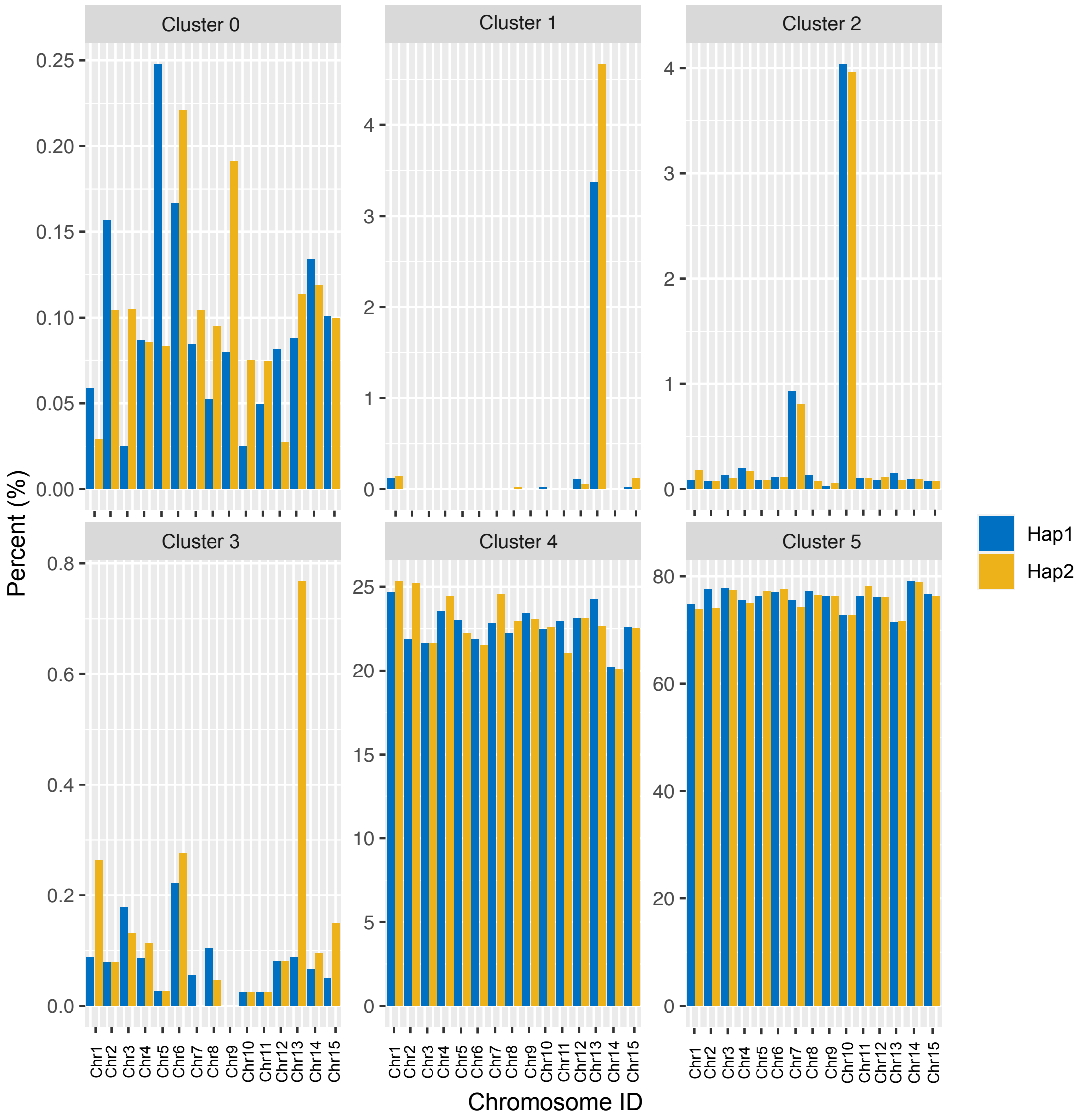

Figure S17. The different proportion of the clusters grouped by GDA results among different chromosomes and haplotypes.

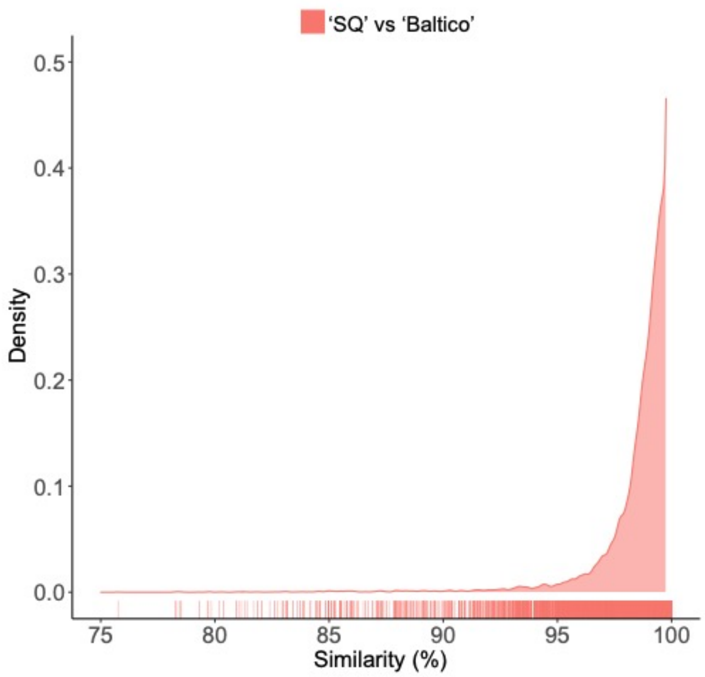

Figure S18. The CDS similarity of between the carnation 'Scarlet Queen' and 'Baltico'.

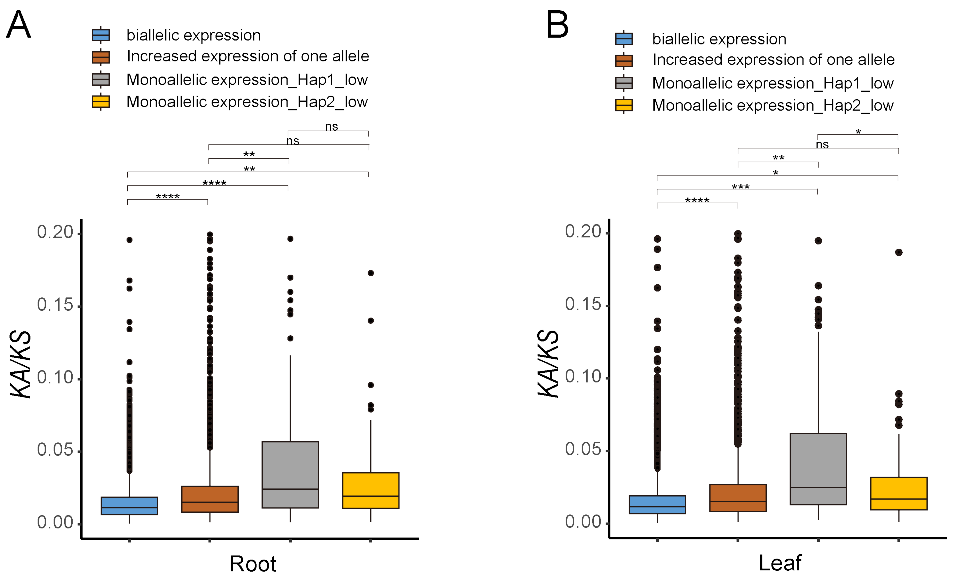

Figure S19. *Ka/Ks* values for different classes of ASE in root (A) and leaf (B). The dots represent Outliers. Boxes represent 25-75% of the value. The upper and lower horizontal lines represent the range with 1.5 Inter Quartile Range (IQR).

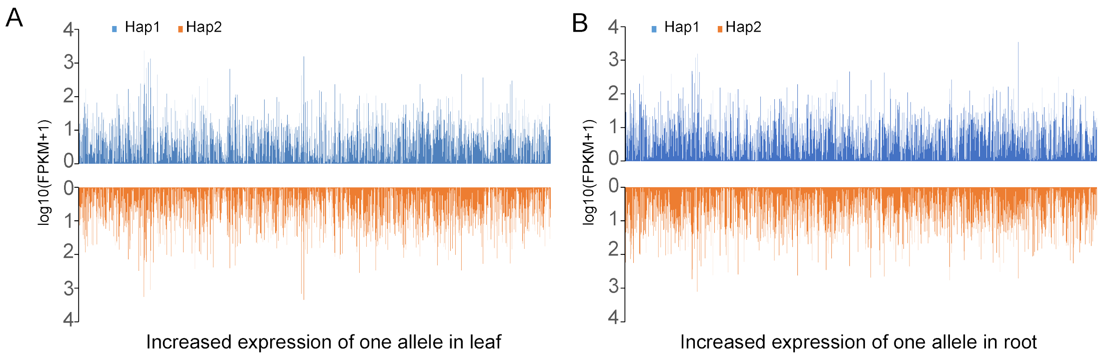

Figure S20. Distribution of ASE (class of ‘increased expression of one allele’) in two haplotypes in leaf (A) and root (B). Expressions are presented as log10(FPKM+1).

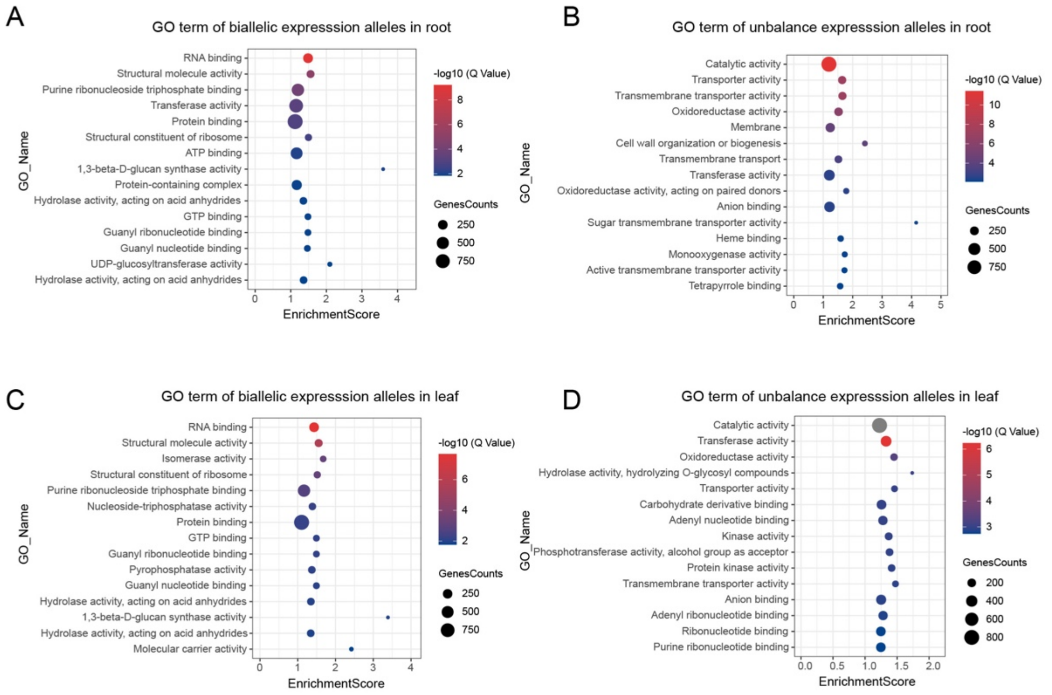

Figure S21. The GO enrichment analysis results of the alleles. (A) GO term of biallelic expression alleles in root. (B) GO term of unbalance expression alleles in root. (C) GO term of biallelic expression alleles in leaf. (D) GO term of unbalance expression alleles in leaf.

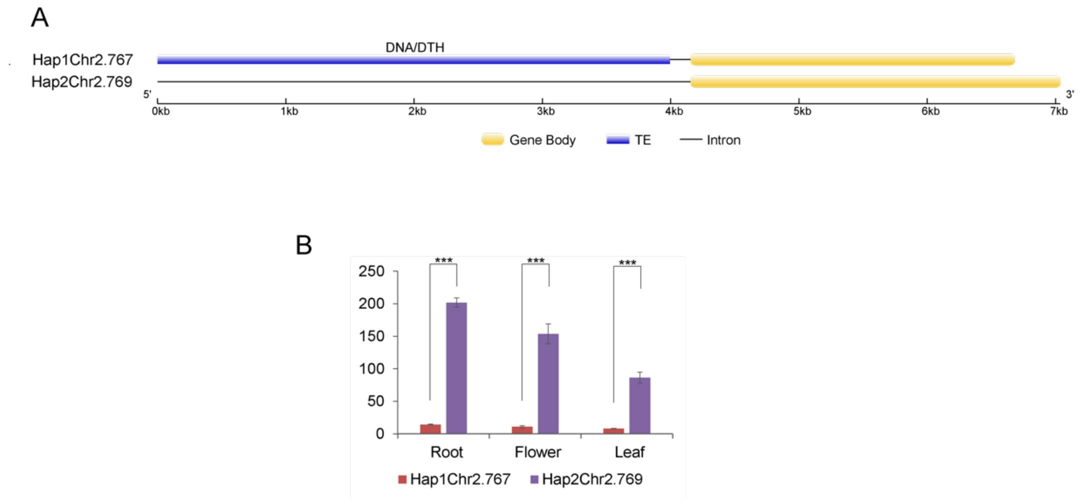

Figure S22. The ASE with specific intact TE insertion. The yellow boxes represent the gene body region, and the lines represent introns and intergenic regions. The bar plot refers to the genes' FPKM.

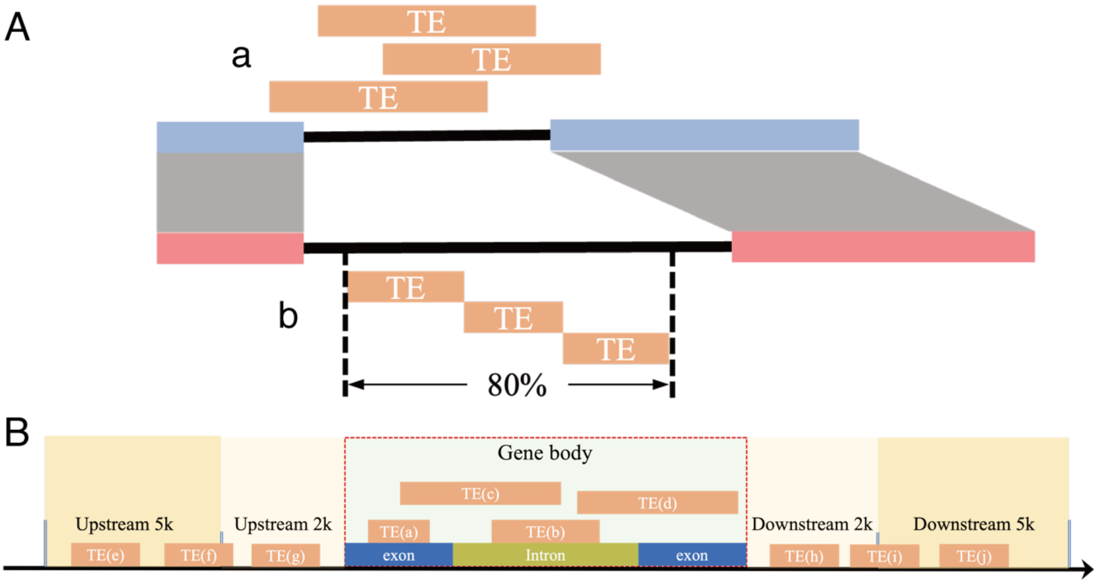

Figure S23. Identifying the types of TEs insertions. (A) we judged the SV (indel and highly divergent regions) were correlated with the TEs if there was a TEs insertions and account for the 80% of total length of this SV (a); and if there were multiple TEs insertions and all these TEs length account for the 80% total length of the SV (b). (B) we classified the TEs insertion position into 5 types. The TEs inserted into the gene body regions (type a-d), and the exon region (type a) and intron region (type b); the TEs inserted into the upstream 5 Kbp regions (type e-g), and upstream 2kbp region (type g); the TEs inserted into the downstream 5Kbp region (type h-j), and downstream 2Kbp (type h).
